# Genome-wide identification, in silico characterization and expression analysis of the RNA helicase gene family in chickpea (*C. arietinum* L.)

**DOI:** 10.1038/s41598-022-13823-9

**Published:** 2022-06-13

**Authors:** Sheel Yadav, Yashwant K. Yadava, Deshika Kohli, Shashi Meena, Gopal Kalwan, C. Bharadwaj, Kishor Gaikwad, Ajay Arora, P. K. Jain

**Affiliations:** 1 ICAR-National Institute for Plant Biotechnology, New Delhi, 110012 India; 2 Division of Plant Physiology, ICAR-Indian Agricultural Research Institute, New Delhi, 110012 India; 3 Division of Genetics, ICAR-Indian Agricultural Research Institute, New Delhi, 110012 India

**Keywords:** Molecular biology, Plant sciences

## Abstract

The RNA helicases are an important class of enzymes which are known to influence almost every aspect of RNA metabolism. The majority of RNA helicases belong to the SF2 (superfamily 2) superfamily, members of which are further categorized into three separate subfamilies i.e., the DEAD, DEAH and DExD/H-box subfamilies. In chickpea, these RNA helicases have not been characterized until now. A genome-wide analysis across the chickpea genome led to the identification of a total of 150 RNA helicase genes which included 50 *DEAD*, 33 *DEAH* and 67 *DExD/H*-box genes. These were distributed across all the eight chromosomes, with highest number on chromosome 4 (26) and least on chromosome 8 (8). Gene duplication analysis resulted in identification of 15 paralogous gene pairs with Ka/Ks values < 1, indicating towards the genes being under purifying selection during the course of evolution. The promoter regions of the RNA helicase genes were enriched in cis-acting elements like the light and ABA-responsive elements. The drought responsiveness of the genes was analysed by studying the expression profiles of few of these genes, in two different genotypes, the cultivated variety ICC 8261 (kabuli, *C. arietinum*) and the wild accession ILWC 292 (*C. reticulatum*), through qRT-PCR. These genotypes were selected based on their drought responsiveness in a field experiment, where it was observed that the percentage (%) reduction in relative water content (RWC) and membrane stability index (MSI) for the drought stressed plants after withholding water for 24 days, over the control or well-watered plants, was least for both the genotypes. The genes *CaDEAD50* and *CaDExD/H66* were identified as drought-responsive RNA helicase genes in chickpea. The protein encoded by the *CaDExD/H66* gene shares a high degree of homology with one of the CLSY (CLASSY) proteins of *A. thaliana*. We hypothesize that this gene could possibly be involved in regulation of DNA methylation levels in chickpea by regulating siRNA production, in conjunction with other proteins like the Argonaute, RNA dependent RNA polymerases and Dicer-like proteins.

## Introduction

In order to acclimatize to the rapidly changing environmental conditions and perturbations, living organisms have to modify and regulate gene expression of various genes, which is facilitated by a diverse array of enzymes. The enzymes involved in nucleic acid metabolism, is one such class of regulatory enzymes. Amongst these, the helicases are an important class of enzymes which play key roles in nucleic acid metabolism including vital cellular processes like replication, recombination, repair, transcription, translation, mRNA splicing and export, etc^1^. The backbone reaction catalysed by these enzymes is the ATP dependent unwinding of a duplex DNA or RNA molecule. The helicases have been categorized into six superfamilies, namely SF1 to SF6^2^. Amongst these the SF2 superfamily is the most well characterized and largest RNA helicase superfamily. Based on sequence variations within one of the highly conserved motifs of these proteins, the DEAD motif or motif II (Asp-Glu-Ala-Asp), the SF2 RNA helicases are broadly categorized into three different subfamilies i.e., the DEAD, DEAH and DExD/H-box subfamilies^3^. These proteins consist of two highly conserved RecA (Recombinase protein) like domains (abbreviated D1 and D2) as parts of a core helicase (~ 200–700 amino acids) and are composed of nine characteristic motifs. The domain D1 consists of motifs Q, I, Ia, Ib, II and III. The domain D2 consists of motifs IV, V and VI. Each of the nine motifs play diverse roles, which are crucial for the enzymatic activity of the helicases such as ATP hydrolysis, RNA binding and mediating intramolecular interactions^1–3^. These helicases even though structurally similar are functionally very diverse. They are involved in different molecular mechanisms governing various aspects of RNA metabolism such as RNA splicing, ribosome assembly, mRNA export, alteration in conformation of ribonucleotides and RNPs, mRNP assembly, etc^4^. They also play important roles during cellular differentiation and organ maturation^5^. Some of these are known to play significant role in cell signaling pathway induced in response to viral infection, by activating interferons and cytokines, through phosphorylation of various transcription factors^6^. Recently, their roles in governing abiotic stress tolerance in plants has been elaborately studied. The AtRH3, 7, 9, 25, AtHELPS, LOS4 in *Arabidopsis thaliana*, OsABP, OsRH17 in *Oryza sativa* and TaRH1 in *Triticum aestivum*, are a few of the abiotic stress-responsive RNA helicases that have been identified^2^. The functional significance of a chloroplast localized DEAD-box helicase, OsRH58 under drought stress has been demonstrated in rice^7^. It was observed that the expression of several chloroplast proteins like rubisco large subunit (RbcL), D1 protein of PSII (PsbA), beta subunit of ATP synthase (AtpB), etc., increased in OsRH58-expressing transgenic *Arabidopsis* plants, which showed enhanced stress tolerance as compared to the wild type plants under dehydration stress. The regulatory role of these helicases is also exemplified by the DEAD-box helicases namely STRS1 and STRS2 (Stress Response Suppressor). These have been shown to bring about epigenetic suppression of stress-responsive genes in *Arabidopsis* by targeting the RNA-directed DNA methylation (RdDM) pathway^8^. The down-regulation of the *STRS1* and *STRS2* genes enhances the expression of stress-responsive genes. Since this pathway relies on small RNAs, the possibility of these helicases in controlling the expression of different small RNAs remains reasonably high. AtRH27, a DEAD-box RNA helicase in *Arabidopsis*, has been shown to interact with the biogenesis components of miRNAs (microRNAs) like RNA binding proteins (DAWDLE, HYPONASTIC LEAVES 1 and SERRATE) and regulates miRNA biogenesis^9^. In a recent study which bolsters the association between miRNAs and DEAD-box helicases, it was reported that the DEAD-box helicases modulate the formation of nuclear organelles, the dicing bodies (D bodies) in *Arabidopsis*, a site where miRNAs are processed^10^.

The SF2 RNA helicases have been identified in several plant species namely *Glycine max*^4^, *Zea mays*^4^, *Oryza sativa*^4^, *Arabidopsis thaliana*^11^, sweet potato^12^, cotton^13^ and tomato^14^. The earliest DEAD-box helicases which were characterized were the *eIF* (eukaryotic translation initiation factor) genes from *Oryza sativa*, *Triticum aestivum*, *Nicotiana tabacum*, etc^15^. There is however no report on genome-wide identification of RNA helicase genes in chickpea (*C. arietinum*), until now. Since chickpea accounts for as high as 15% of the global pulse production annually, it is a very important pulse crop in terms of ensuring food sustainability, in times to come^16^. In order to increase its productivity, genomics assisted breeding technologies are being rampantly used by breeders to develop new and improved chickpea varieties. Terminal drought tolerance in chickpea has been a trait targeted for improvement, since the incidence of drought at the post flowering stage is estimated to cause yield losses as high as 50%^17^. To understand the molecular basis of drought tolerance, it is important to identify genes which are differentially expressed in response to drought stress in chickpea. Identification of proteins and enzymes which are the primary effector molecules, which in turn go on to modulate stress-responsive gene expression and control a multitude of metabolic pathways by regulating RNA biogenesis and metabolism, therefore is extremely important. Since the RNA helicases are involved in a diverse array of molecular functions, including abiotic stress tolerance, their identification and characterization in chickpea will be of high relevance and utility for chickpea improvement.

The present study was thus undertaken with the objectives of genome-wide identification of the RNA helicase genes in chickpea, their structural characterization (in silico) and analysing their expression in response to drought stress conditions.

## Materials and methods

### Genome-wide identification of RNA helicase genes in chickpea

The *Arabidopsis thaliana* RNA helicase gene and protein sequences were obtained from TAIR (TAIR10 release; https://www.arabidopsis.org/). A local nucleotide and protein database of annotated chickpea genes and proteins, respectively, was created using NCBI command-line tools, BLAST+. For this purpose, the nucleotide and protein sequence files of chickpea (CDC Frontier genome Cav1.0, assembly ASM33114v1) were retrieved from the genome assembly database (NCBI RefSeq). A BLASTP and TBLASTN search was performed against the chickpea protein and gene sequence databases, respectively, using all the known *Arabidopsis* RNA helicase protein sequences as queries, with an e-value cut-off set at 1e-004. The identified proteins were confirmed for the presence of the core helicase domain (PF00271; https://pfam.xfam.org/) by searching them against the NCBI CD (Conserved Domain) database (http://www.ncbi.nlm.nih.gov/Structure/cdd/wrpsb.cgi) and SMART (Simple Modular Architecture Research Tool) database (http://smart.embl-heidelberg.de/). The proteins which did not contain the characteristic domains were removed. Also the proteins which were less than 100 amino acids long, were excluded from further analysis. Based on the sequence conservation at the motif II and the phylogenetic relationship with the known DEAD, DEAH and DExD/H-box proteins of *A. thaliana* and *O. sativa*^4^, the identified RNA helicases in chickpea were categorized into CaDEAD, CaDEAH and CaDExD/H-box proteins, where Ca stands for the *C. arietinum* species. The genomic and cDNA sequences of the identified proteins were acquired from the chickpea genome assembly database.

### Chromosomal localization and gene structure analysis

The physical locations of the RNA helicase genes on the chickpea chromosomes were extracted manually from the genomic database at NCBI. The distribution of the genes across the chromosomes was visualized using MapChart tool^18^. The gene structures of the genes were determined by comparing the coding sequences with the corresponding full-length gene sequences, using the Gene Structure Display Server^19^.

### Synteny and gene duplication analysis

To infer the syntenic relationship of the RNA helicase genes in different species (*Arabidopsis thaliana*, *Cajanus cajan, Cicer arietinum*, *Glycine max* and *Medicago truncatula*), an analysis using MCScanX algorithm was performed and plots were generated using the Dual Synteny Plotter function in TBtools v 1.098693^20^. The duplicated or paralogous gene pairs, were identified using MCScanX algorithm and visualized as a Circos plot. The Ka/Ks (non synonymous/synonymous substitution rates) ratios of the duplicated gene pairs were calculated to study the molecular evolutionary rates for each gene pair. The divergence time of these gene pairs was estimated using the formula “t = Ks/2*λ*”, with *λ* value of 6.05 × 10^−9^ substitutions/synonymous site/year representing neutral substitution^21^. This was expressed in million years ago (Mya).

### Promoter sequence analysis

Promoter sequences (1000 bp upstream from the predicted transcription start site, TSS) of few of the helicase (8 *CaDEAD*, 4 *CaDEAH* and 8 *CaDExD/H*) genes were subjected to search for cis-regulatory elements in the PlantCARE (http://bioinformatics.psb.ugent.be/webtools/plantcare/html/) database. The elements were visualized along the lengths of promoter sequences through TBtools v 1.098693^20^. In addition, the promoter sequences were manually scanned for various, well-characterized, drought-responsive cis-acting elements like the dehydration-responsive element (DRE), G-Box, MYBL, etc. based on a published report where these cis-acting elements have been identified in *GmRD26* gene in soybean^22^.

### In silico characterization of the RNA helicase proteins

The identified proteins were analysed for various physiochemical properties like protein length (number of amino acids), molecular weight (Da) and theoretical isoelectric point (pI), using the online ExPASy website (http://web.expasy.org/)^23^. The Cello v2.5 webserver was used for the prediction of the subcellular location of these proteins, in silico (http://cello.life.nctu.edu.tw/). MEME (Multiple Em for Motif Elicitation, version 4.11.4) was used to identify conserved motifs within the chickpea helicase proteins using the following input parameters: optimum motif width set to ≥ 6 and ≤ 50; maximum number of motifs: 10 (http://meme-suite.org/tools/meme)^24^. Network analysis was performed to determine the physical interaction between the RNA helicase proteins using the STRING database (https://string-db.org/). Since these proteins are known to modulate different aspects of RNA metabolism, their interaction with chickpea small RNA biogenesis and processing proteins like Argonaute (AGO) and RNA dependent RNA polymerase (RDR) was examined^25^. For this purpose, the protein sequences of these genes were retrieved from protein database of NCBI.

### Sequence alignment and phylogenetic tree analysis

A total of 151 DEAD, 106 DEAH and 203 DExD/H-box proteins belonging to three different species (*Arabidopsis thaliana*, *Oryza sativa* and *Cicer arietinum*) were analysed for phylogeny. The protein sequences for *Arabidopsis* and rice RNA helicases were obtained from the previously published report^4^. The sequences were aligned through multiple sequence alignment (MSA) using Muscle tool (http://www.drive5.com/muscle). MEGA7 was used to construct the phylogenetic tree with bootstrapping for 1000 replicates and with the following main parameters: p distance and pairwise deletion (https://www.megasoftware.net/).

### Analysis of gene expression levels of the *CaDEAD*, *CaDEAH* and *CaDExD/H*-box genes

Two sets of publicly available transcriptome data were used to analyse the gene expression profiles of the chickpea RNA helicase genes. The first dataset consisted of the FPKM (Fragments per kilobase of transcript per million mapped reads) values available at the NCBI GEO datasets, for the BioProject ID: PRJNA401922. These were generated from the RNA-Seq data for different tissues (leaves, roots, flowers and young pod) of the genotype, ICC 4958 (cultivated, *C. arietinum*) and leaves of wild chickpea accession, PI 489777 (*C. reticulatum*)^26^. The FPKM values were log 2 transformed for the *CaDEAD, CaDEAH* and *CaDExD/H* loci. The heatmap depicting the differential expression of the genes was drawn by the software TBtools v 1.098693^20^.

The second dataset corresponded to the log 2 FC values of gene expression derived from the RNA-Seq dataset for the leaf tissues, collected at the shoot apical meristem stage, of drought tolerant (DT, ICC 8261) and drought sensitive (DS, ICC 283) genotypes, both belonging to the cultivated species, *C. arietinum* (BioProject ID: PRJNA413294). Heatmap was constructed by the software TBtools v 1.098693^20^ using the log 2 FC values for the *CaDEAD, CaDEAH* and *CaDExD/H*-box genes.

### Plant materials and drought stress treatment

Seven different genotypes of chickpea, six (ICC 4958, ICC 8261, Pusa 362, SBD 377, ICC 1882, ICC 283) belonging to the cultivated species, *C. arietinum* and one (ILWC 292) belonging to the wild species, *C. reticulatum* were grown in completely randomized block design during Rabi 2020–2021 (Nov-March), under a rain out shelter (ROS) at the Indian Agricultural Research Institute (IARI), Pusa, New Delhi (28° 38′ N 77° 08′ E). The temperature during the day was ~ 25 °C and ~ 15 °C during the night, at the time of sowing. The seeds of these genotypes were obtained from the Pulse Research Laboratory, Division of Genetics, IARI (Supplementary Fig. S1). ILWC 292 is a registered cyst nematode-resistant germplasm and is a wild collection in the public repository of ICARDA, Aleppo, Syria. The voucher specimen is maintained by ICARDA^27^. These seven genotypes were selected based on their differential drought tolerance with ICC 4958, ICC 8261, Pusa 362 and ILWC 292 being drought tolerant (DT) and SBD 377, ICC 1882 and ICC 283 being drought sensitive (DS). Black coloured polypropylene bags (50 × 30 cm) were used for sowing the seeds. Two holes each of 1 cm diameter were cut out at the bottom of the bags in order to allow excess water to drain from the bags. Each bag was filled with equal amounts of soil collected from the nearby chickpea cultivation field of IARI. The soil was prepared as per the standard agronomic practices used for chickpea cultivation. Two seeds/genotype were sown at a depth of 2 cm in the soil. A day prior to sowing, the seeds were treated with the fungicide Bavistin @ 5 g/kg of seed and *Rhizobium* culture @ 25 g/kg of seed. A total of nine bags were sown per genotype (1 genotype × 3 replicates × 3 treatments—Control, T12, T24) amounting to a total of 63 bags (7 genotypes × 9 bags) (Supplementary Fig. S2). Initially, for the first 90 days after sowing (90 DAS) all the plants were watered with equal amounts of water regularly in order to roughly maintain same levels of soil moisture in all the bags. At 93 days after sowing (93 DAS, after the initiation of flowering in most of the genotypes), the bags were separately labelled (Supplementary Fig. S3). One-third of the bags were designated as control (C) bags. Plants growing in these bags were regularly watered (with equal amounts of water) throughout the experimental period (117 days). One-third of the bags were labelled as T12, the plants growing in these bags were not supplied with water for 12 days, beginning with 93 DAS. The remaining third of the bags were labelled as T24 and the plants growing in these bags were not supplied with any water for 24 days post 93 DAS.

The leaf tissues of all the plants (regularly watered until now) were collected for physiological characterization on day 0 (93 + 0 = 93 DAS). On day 12 (93 + 12 = 105 DAS), leaf tissues were collected separately from C12 and T12 plants and on day 24 (93 + 24 = 117 DAS), sampling for leaf tissues was done separately from the C24 and T24 plants. For the purpose of gene expression analysis, the leaf samples were collected in liquid nitrogen and stored at − 80 °C for RNA isolation.

### Physiological and root morphological characterization

The leaf relative water content (RWC) of chickpea genotypes was estimated at intervals of day 0 (day of drought stress initiation; 93 DAS), day 12 (105 DAS) and day 24 (117 DAS) after drought stress initiation. The RWC was estimated for five biological replicates/genotype/treatment. For this purpose, third fully expanded, young leaves from the top were collected in polybags before noon from the plants and kept on ice packs. These were collected for all the plants on day 0 and both the control and drought stressed plants separately, on days 12 and 24. The fresh weights (FW) of these leaves were recorded. These were then kept in covered petriplates containing distilled water for 4 h and then weighed to obtain the turgid weights (TW). These samples were thereafter dried at 65 °C in an oven for 24–48 h and the dry weights (DW) of the samples were recorded. The RWC (%) values were calculated according to the formula given below^28^:

$${\text{RWC }}\left( \% \right) \, = \{ \left( {{\text{FW}} - {\text{ DW}}} \right)/\left( {{\text{TW}} - {\text{ DW}}} \right)\} \times {1}00.$$

The sampling of leaves for estimation of membrane stability index (MSI) was done along with the sampling for RWC. For estimation of MSI, 500 mg fresh leaf samples were collected for each genotype. The leaf samples were thoroughly washed 2–3 times with de-ionized water. These were then kept in test tubes containing 10 ml of distilled water. The test tubes were kept in a water bath at 40 °C for 30 min and conductivity of the medium (C1) was recorded by using a conductivity meter (EutechCyberScan CON 110). After recording the C1 values, the test tubes were again kept in water bath at a temperature of 100 °C for 15 min. At the end of 15 min, the tubes were taken out and allowed to cool to room temperature. The conductivity of the samples was recorded again (C2). The MSI (%) was calculated using the following formula^29^:

$${\text{MSI }}\left( \% \right) \, = { 1} - \left( {{\text{C1}}/{\text{C2}}} \right) \times {1}00.$$

At the end of the experimental period, 117 DAS, the roots of the C24 and T24 plants, for each genotype, were carefully extracted from the soil and washed thoroughly. These were then suspended in a transparent tray filled with water (2–3 mm ht). For the purpose of root scanning the image analysis system (WinRhizo, Regent Instruments INC., Quebec, Canada) was used. The roots were carefully disentangled and data were recorded for various root traits namely root length (RL, cm), surface area (SA, cm^2^), root diameter (RD, mm) and root volume (RV, cm^3^). Root scanning was performed for three biological replicates/genotype/treatment.

### Identification of the drought-responsive *CaDEAD, CaDEAH* and *CaDExD/H*- box genes through qRT-PCR

Total RNA was isolated from frozen leaf tissues of the samples (ICC 8261 and ILWC 292, C24 and T24 for each genotype, 117 DAS) using the Spectrum™ Plant Total RNA Kit (Sigma Life Science). These two genotypes were chosen based on their favourable response to drought stress and also considering that since there exists greater diversity (in terms of SNPs) between the kabuli and wild genomes of chickpea^30,31^, higher levels of differences in gene expression might be observed for the two genotypes as opposed to gene expression level differences existent between the desi and wild genotypes. The isolated RNA was eluted in the supplied elution buffer (EB). The quantity and quality of the isolated RNA was estimated using Nanodrop spectrophotometer (NanoDrop™ Lite Spectrophotometer, Thermo Fisher Scientifc). The RNA was converted into cDNA by using the cDNA synthesis kit (Invitrogen™SuperScript™ III First-Strand Synthesis System) following the manufacturer’s instructions. Real-time quantitative PCR (qRT-PCR) was performed using SYBR Green mix (TaKaRa) on a Real Time PCR Thermo Cycler (EppendorfRealPlex 2 qPCR). The thermal cycling conditions used were: 94 °C for 10 min, 40 cycles with each cycle consisting of 94 °C for 30 s and 60 °C for 15 s, followed by melting curve analysis. The gene expression analysis was carried out for 7 *CaDEAD*, 1 *CaDEAH* and 7 *CaDExD/H*- box genes. Primers were designed using the Primer Quest tool (https://www.idtdna.com). All the primers for qRT-PCR are listed in Supplementary Table S1. These genes were selected based on their phylogenetic relationships and an attempt was made to select genes with higher diversity. The experiment was performed for 36 samples (2 genotypes × 2 treatments (C24 and T24) × 3 biological replicates × 3 technical replicates). The relative fold differences were calculated based on the comparative Ct method using the 2−△△Ct method^32^, using the *GAPDH* gene (Glyceraldehyde-3-phosphate dehydrogenase, GenBank accession no. AJ010224) as an internal reference gene. The log2 FC values were derived for four different combinations i.e., ILWC 292 drought stressed T24 plants vs ICC 8261 drought stressed T24 plants. The other combinations were ILWC 292 C24 plants vs ICC 8261 C24 plants; ILWC 292 C24 vs T24 and ICC 8261 C24 vs T24. For these combinations, the expression levels of the genes for ILWC 292 T24, ILWC 292 C24, ILWC 292 C24 and ICC 8261 C24, respectively, were considered as control and normalized to 1. This was done to identify genotype-specific and treatment induced differences in gene expression of the genes in response to drought stress. The specificity of the primers was checked by loading the amplified products on a 1.5% agarose gel (Supplementary Fig. S4). BLASTN searches were also performed for the 15 gene sequences (*C. arietinum*) against the *C. reticulatum* PI489777 v2.0 genome to confirm the specificity of genes amplified (https://www.pulsedb.org/).

### Statistical analysis

The data for the physiological parameters (RWC and MSI) and root traits were subjected to statistical analysis (https://www.socscistatistics.com). For each trait, the mean values and coefficient of variation (CV) were calculated (Supplementary Table S2). One-way analysis of variance (ANOVA) with post-hoc Tukey HSD (Honestly significant difference) was performed to test for statistical significance between means of different traits for the genotypes studied. Student’s t-test was conducted in order to study the statistical significance of the differences in means of the control and treated plants for all the physiological, root morphological and qRT-PCR data.

### Ethical approval

The authors declare that the experimental research work involving the growth of plants in this study, was conducted in compliance with relevant institutional, national, and international guidelines and legislation.

## Results

### The chromosomal distribution of the RNA helicase genes across the chickpea genome and their structural characterization

**Figure 1:**
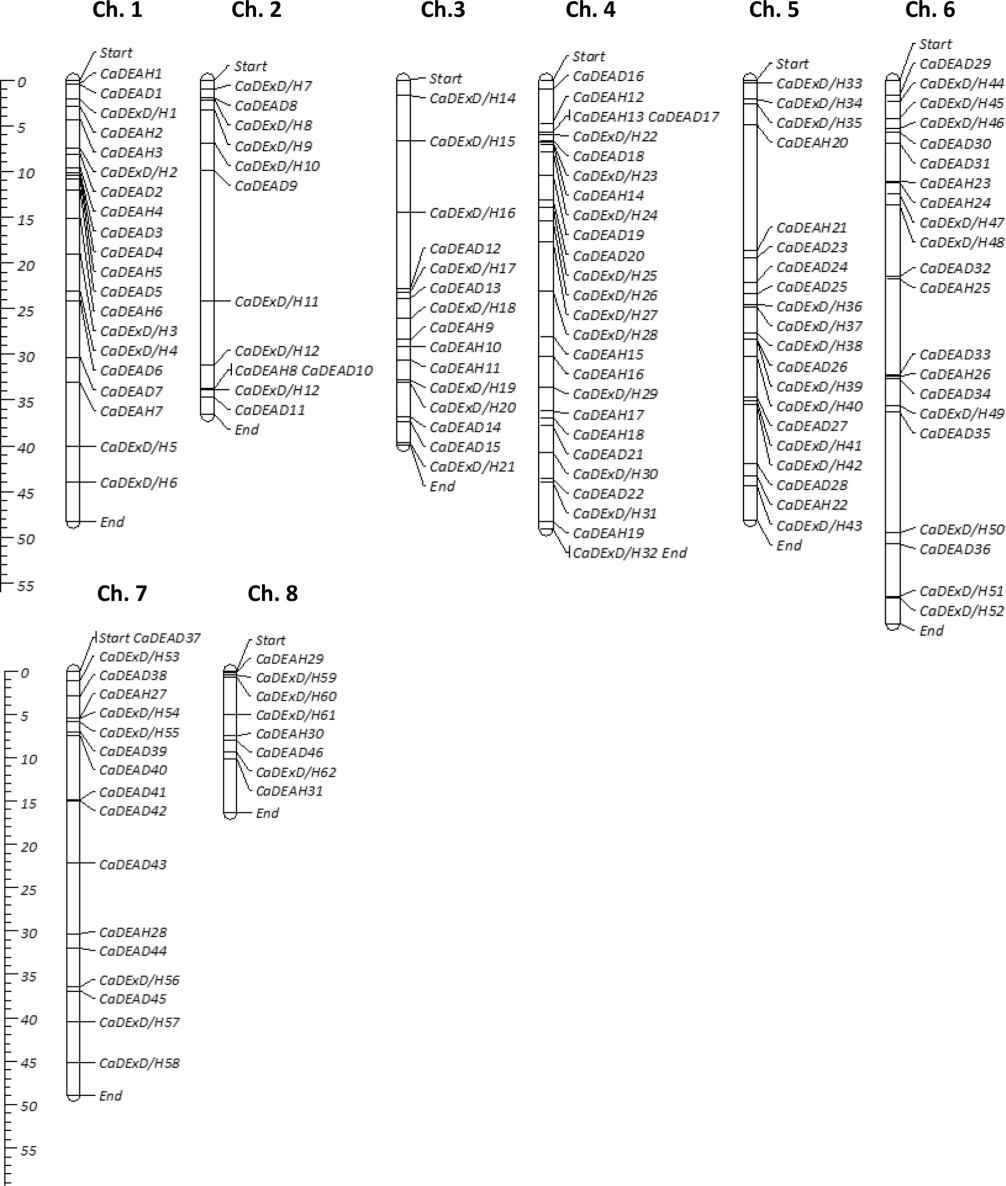
The distribution of the RNA helicase genes (139 in number) across the eight chickpea chromosomes. The genes were mapped as per the physical location (in Mega bases, Mb) on the chromosomes and the gene name is indicated on the right side of each bar. The scale on the left is in Mb. Eleven genes which were mapped on the unplaced scaffolds are not shown here.

Based on combined results of the BLAST searches (BLASTP and TBLASTN), a total of 150 RNA helicase genes could be identified in the chickpea genome. These were classified into three different subfamilies i.e., the *CaDEAD* (50), *CaDEAH* (33) and *CaDExD/H* (67)*-*box genes, where the numbers in parenthesis indicate the number of genes identified in each subfamily. The genes belonging to each of the three subfamilies were chronologically named based on their chromosomal positions in congruence with the previously published reports in tomato, cotton, etc^13,14^. These genes were unevenly distributed across all the eight chromosomes and a few were mapped on the unplaced scaffolds of the assembly (Supplementary Table S3). The highest number of genes were present on chromosome 4 (26) and the least on chromosome 8 (8). Chromosome 4 also had the highest density of the RNA helicase genes (52%) whereas chromosome 2 had the least density of the genes (32%) (Fig. 1). A total of 11 genes were mapped on the scaffolds.

**Figure 2:**
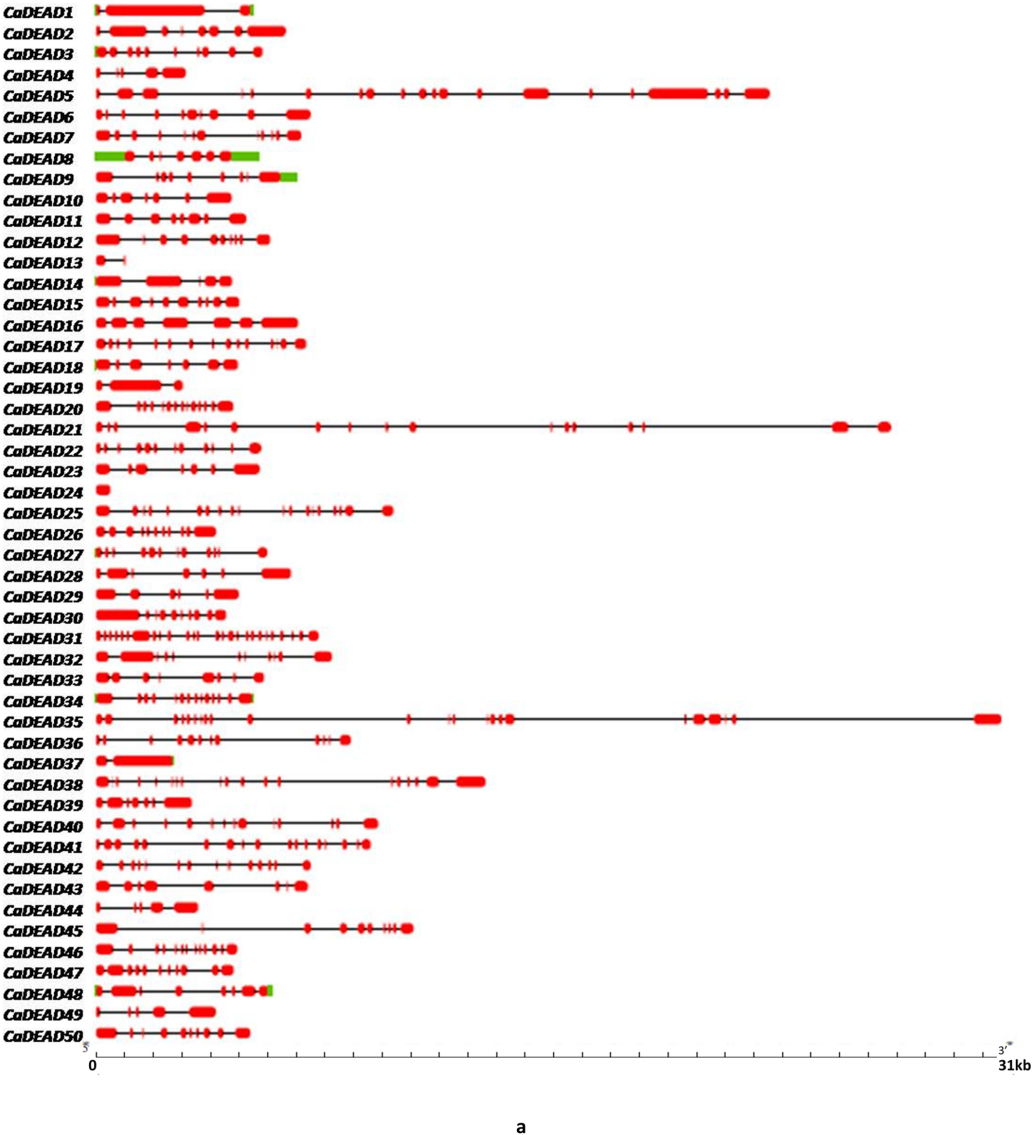

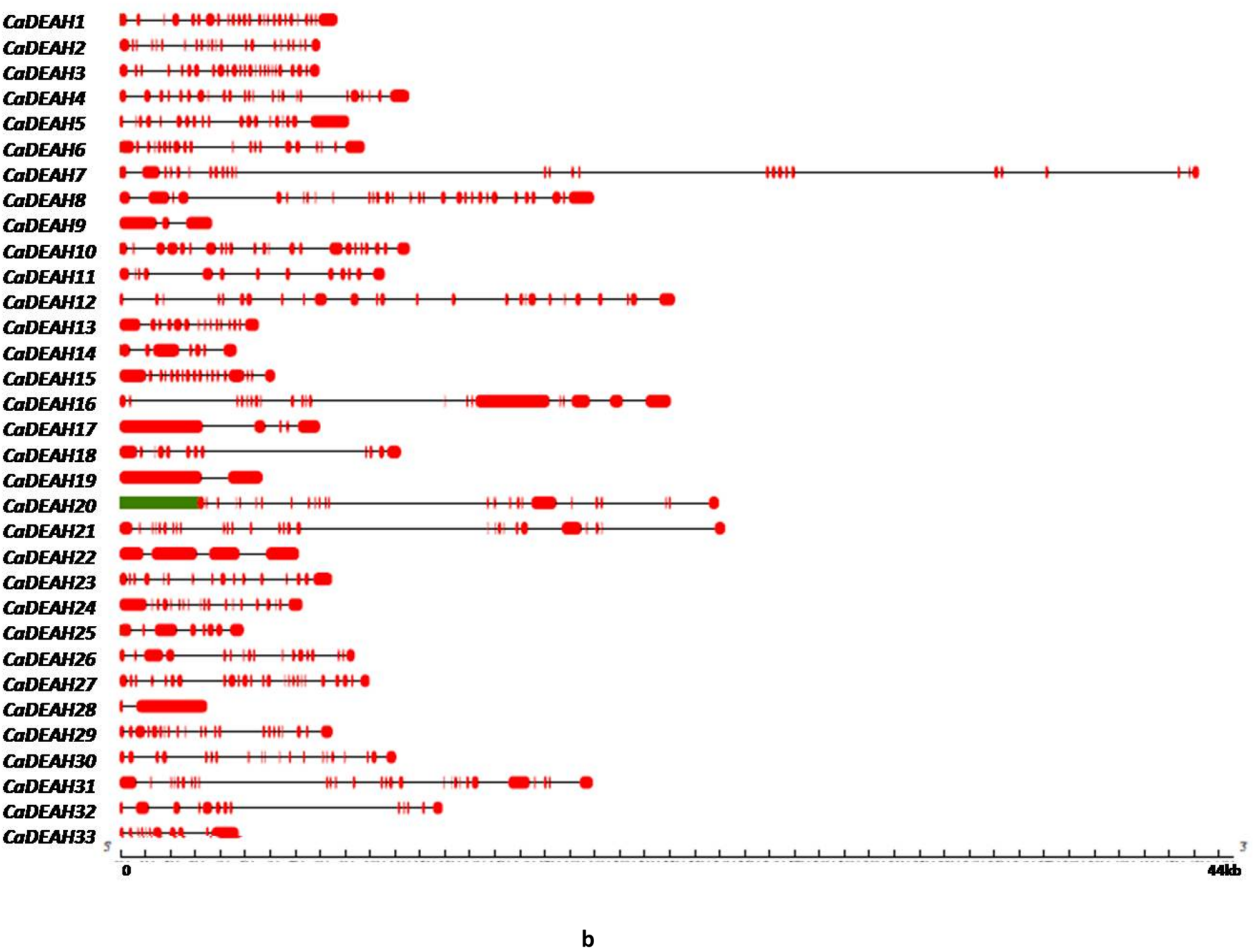

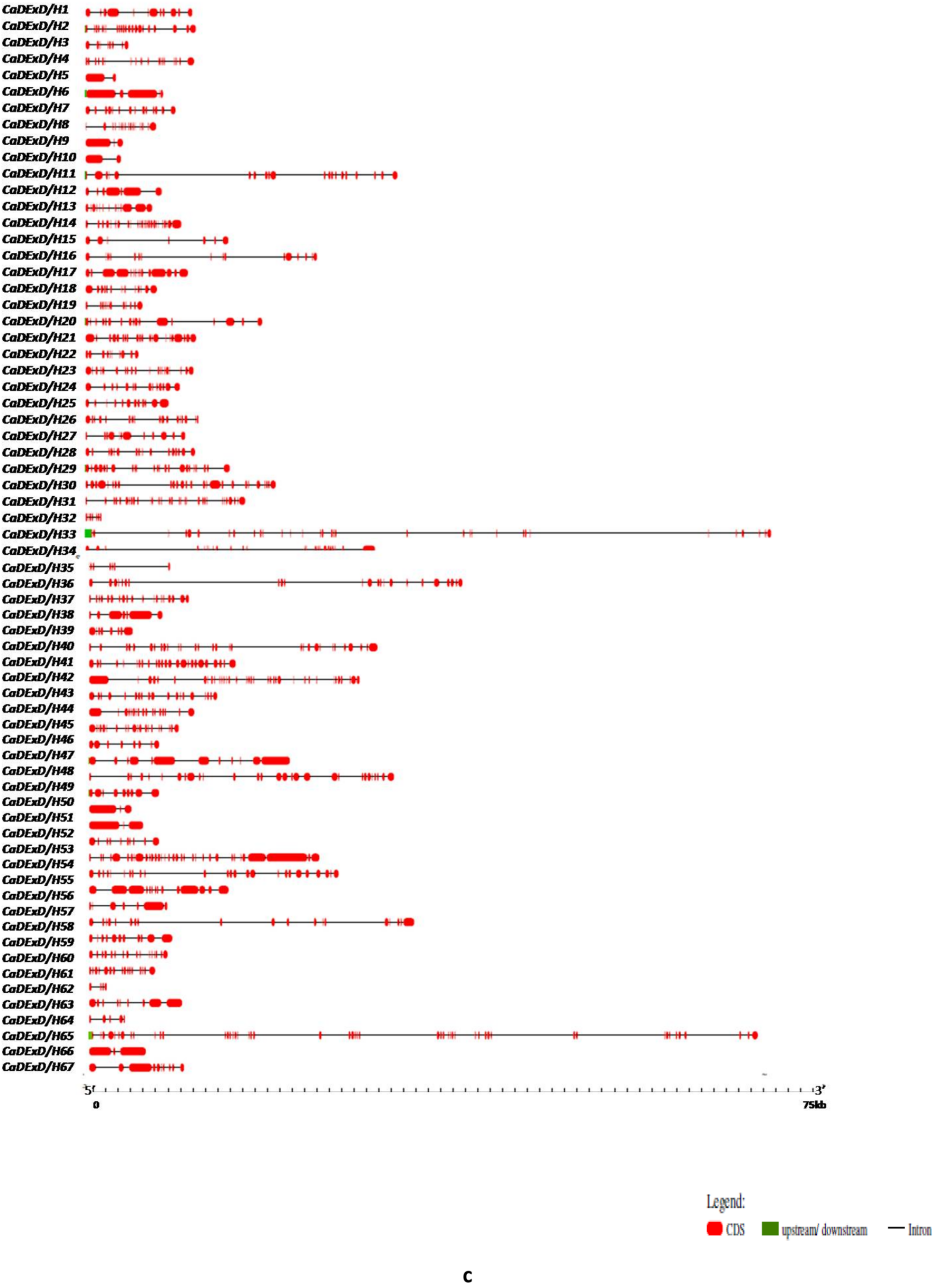
The gene structures depicting the exon–intron organization of the RNA helicase genes in chickpea. The scales at the bottom of each panel depict the gene lengths in Kilo bases (Kb). (**a**) The *CaDEAD*-box genes. (**b**) The *CaDEAH*-box genes. (**c**) The *CaDExD/H-*box genes.

The smallest gene amongst the *CaDEAD-*box genes was the *CaDEAD24* gene (506 bp) which is a putative eukaryotic translation initiation factor (eIF) gene while the longest gene was *CaDEAD35* (31,471 bp)*.* For the *CaDEAH* subfamily the smallest member was *CaDEAH28* gene (3622 bp) followed closely by *CaDEAH9* gene (3824 bp), while the longest member was *CaDEAH7* (44,560 bp). The smallest gene within the *CaDExD/H* gene subfamily was *CaDExD/H62* (1511 bp) and the longest gene was *CaDExD/H33* (74,802 bp) followed by *CaDExD/H65* (58,276 bp). The number of introns in all the three subfamilies of RNA helicases was highly variable. The number of introns for the *CaDEAD* gene subfamily ranged from as low as 0 (*CaDEAD24*) to 24 (*CaDEAD31*) (Fig. 2a)*.* The gene *CaDEAH28* possessed only 1 intron whereas the genes *CaDEAH1* and *CaDEAH3* possessed as high as 24 and 23 introns, respectively (Fig. 2b). The genes *CaDExD/H5* and *CaDExD/H10* possessed 1 intron each whereas the gene *CaDExD/H54* possessed as many as 33 introns (Fig. 2c).

### Syntenic relationship of the chickpea RNA helicase genes with other plant species and identification of paralogous gene pairs within the chickpea genome

In order to determine the evolutionary relationship of the chickpea RNA helicase gene family with other plant species, we generated and analyzed the synteny maps between chickpea and the model plant species, *A. thaliana* and other leguminous species like *M. truncatula*, *C. cajan* and *G. max* (Supplementary Fig. S5). A total of 39 RNA helicase loci showed syntenic relationship with 46 loci in *A. thaliana*. A higher degree of synteny was observed for the RNA helicase genes of chickpea and the legumes studied. 87 chickpea RNA helicase genes were syntenic with 114 genes from *M. truncatula.* 53 genes of chickpea RNA helicase gene family had 64 corresponding syntenic genes in *C. cajan*. For *G. max*, 85 genes in chickpea were syntenic with as many as 181 genes*.*

**Figure 3:**
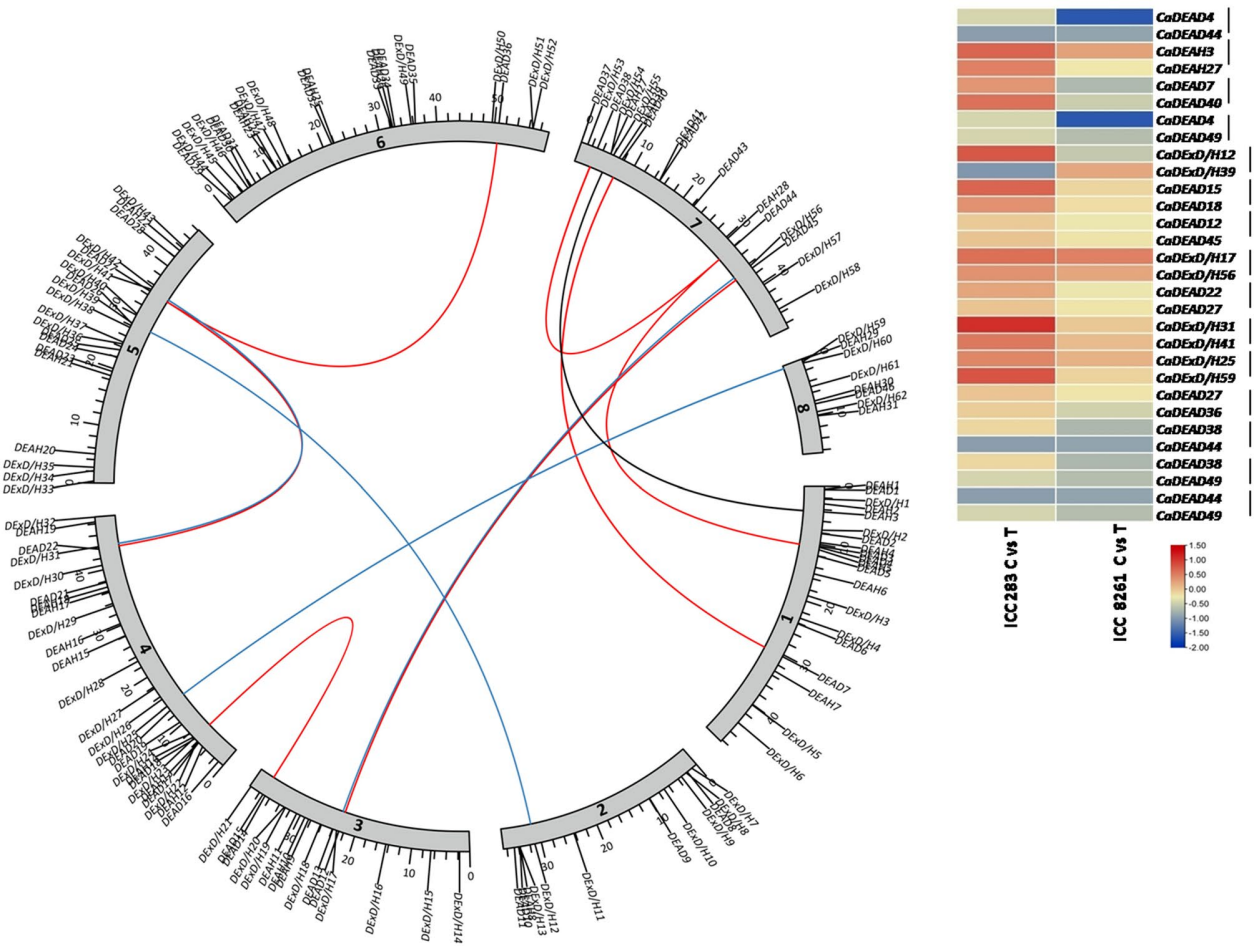
The distribution of duplicated RNA helicase gene pairs across the chickpea chromosomes. The red, black and blue lines in the Circos plot connect the duplicate *CaDEAD*, *CaDEAH* and *CaDExD/H*-box genes, respectively. These are 12 in number as the gene pairs (3) located on scaffolds are not represented. A total of 15 duplicate gene pairs were identified, whose expression levels in terms of log2 FC on exposure to drought stress treatment, derived from publicly available RNA-Seq data of leaf tissues from two contrasting genotypes, ICC 8261, drought tolerant and ICC 283, drought sensitive, are shown as heatmap. Vertical lines connect the genes constituting the duplicated gene pair.

A total of 15 paralogous gene pairs were identified for the RNA helicase gene family in chickpea (Table 1). The highest number of gene duplication events were observed for the *CaDEAD* genes (10/15) and least (1/15) for the *CaDEAH* genes. Eleven of these duplication events could be ascribed to segmental duplication as the duplicated genes for these gene pairs were present on different chromosomes. One gene pair, *CaDEAD38*/*CaDEAD44* was categorized as a proximal duplication event as they are proximally placed on the same chromosome but possibly separated by a few intervening non-homologous genes^33^. For three gene pairs, where one of the genes were mapped on the scaffolds, the nature of duplication could be segmental, tandem or proximal. The Ka/Ks ratios for all the gene pairs were < 1, indicating the existence of negative selective pressure or purifying selection for these genes. In terms of gene expression, almost all of the 15 gene pairs showed similar digital expression levels in the two contrasting genotypes, ICC 8261, the DT genotype and ICC 283, the DS genotype, on exposure to drought stress treatment. Only for one gene pair namely *CaDExD/H12/CaDExD/H39* the expression profiles of the two genes were different in both the genotypes on drought stress exposure (Fig. 3).

**Table 1:** Paralogous gene pairs for the RNA helicase gene family identified in chickpea.

S. no.	Gene 1	Gene 2	Chromosome localization	Ka	Ks	Ka/Ks	Time (Mya)	Duplication event
1	*CaDEAD4*	*CaDEAD44*	1, 7	0.04	0.60	0.06	49.63	Segmental
2	*CaDEAH3*	*CaDEAH27*	1, 7	0.06	0.78	0.08	64.21	Segmental
3	*CaDEAD7*	*CaDEAD40*	1, 7	0.18	0.61	0.29	50.58	Segmental
4	*CaDEAD4*	*CaDEAD49*	1, US	0.06	1.61	0.03	132.80	Unknown
5	*CaDExD/H12*	*CaDExD/H39*	2, 5	0.31	1.01	0.30	83.34	Segmental
6	*CaDEAD15*	*CaDEAD18*	3,4	0.22	0.75	0.29	62.18	Segmental
7	*CaDEAD12*	*CaDEAD45*	3, 7	0.06	0.60	0.09	49.96	Segmental
8	*CaDExD/H17*	*CaDExD/H56*	3, 7	0.13	0.54	0.25	44.43	Segmental
9	*CaDEAD22*	*CaDEAD27*	4, 5	0.02	0.65	0.03	53.87	Segmental
10	*CaDExD/H31*	*CaDExD/H41*	4, 5	0.14	0.56	0.25	46.58	Segmental
11	*CaDExD/H25*	*CaDExD/H59*	4, 8	0.10	0.55	0.17	45.32	Segmental
12	*CaDEAD27*	*CaDEAD36*	5, 6	0.06	2.21	0.03	182.74	Segmental
13	*CaDEAD38*	*CaDEAD44*	7, 7	0.07	1.98	0.04	163.58	Proximal
14	*CaDEAD38*	*CaDEAD49*	7, US	0.04	0.56	0.06	46.55	Unknown
15	*CaDEAD44*	*CaDEAD49*	7, US	0.06	2.27	0.02	187.56	Unknown

*US* unplaced scaffolds, *Ka* nonsynonymous substitutions per nonsynonymous site, *Ks* synonymous substitutions per synonymous site, *T* approximate time (Mya; million years ago) of the duplication event.

### The cis-acting elements within the promoter regions of the selected *CaDEAD, CaDEAH* and *CaDExD/H-*box genes

**Figure 4:**
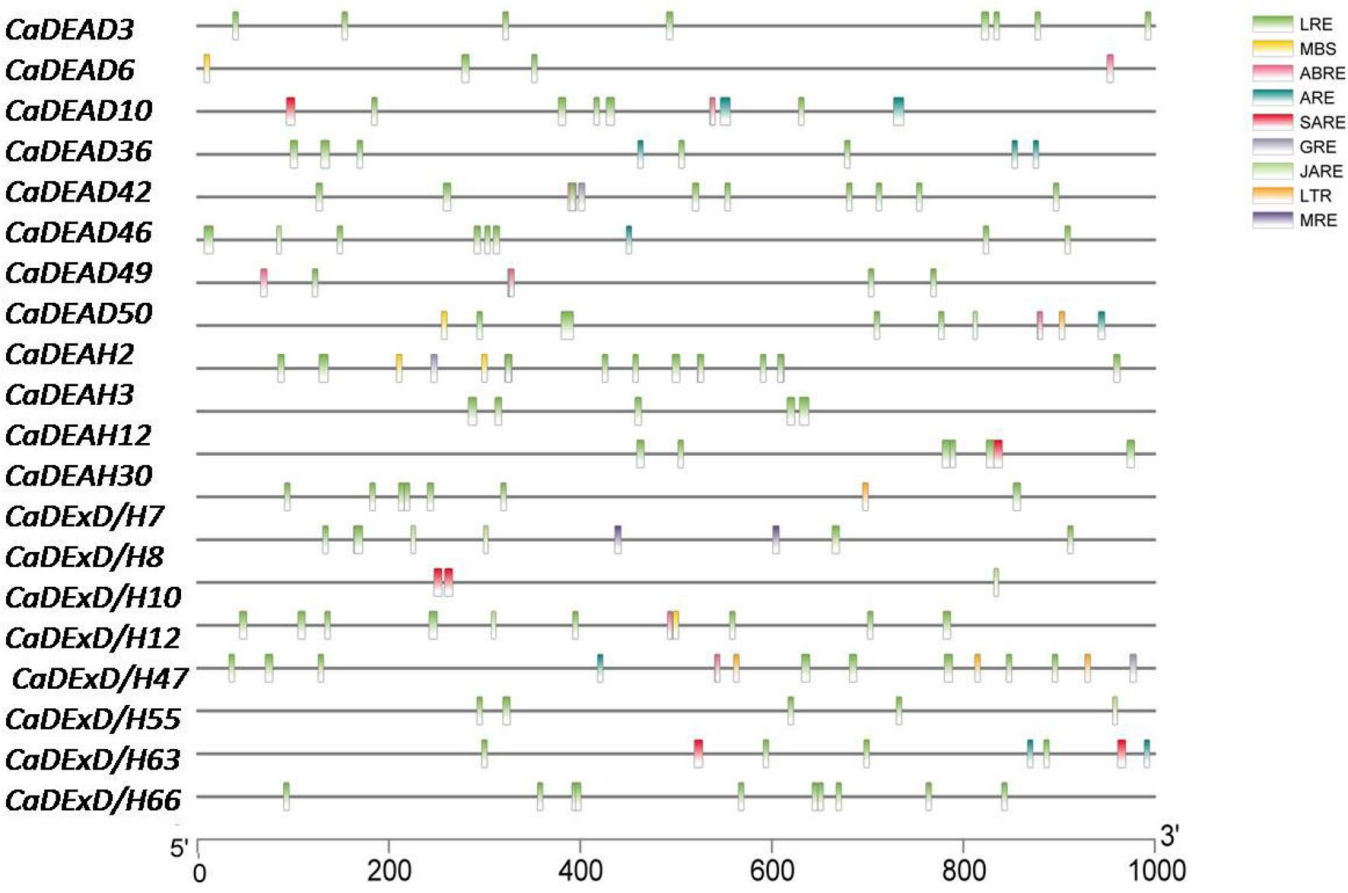
The cis-acting elements in the promoter regions of selected RNA helicase genes (8 *CaDEAD* + 4 *CaDEAH* + 8 *CaDExD/H-*box) genes in chickpea. The scale at the bottom represents 1000 bp upstream from the TSS for the genes. The elements identified are abbreviated as *LRE* light responsive-element, *MBS* MYB binding site involved in drought-inducibility, *ABRE* element involved in the abscisic acid responsiveness, *ARE* part of an auxin-responsive element, *SARE* element involved in salicylic acid responsiveness, *GRE* gibberellin-responsive element, *JARE* element involved in the methyl jasmonate or MeJA responsiveness, *LTR* element involved in low-temperature responsiveness and *MRE* MYB binding site involved in light responsiveness.

The cis-acting elements in the promoter regions of the genes serve as binding sites for transcription factors. The identification of these elements therefore could help in predicting the potential function of these genes. The cis-acting elements present in 1000 bp upstream regions from the transcription start site (TSS) of 20 RNA helicase genes were identified. The genes included 8 *CaDEAD*, 4 *CaDEAH* and 8 *CaDExD/H*- box genes. An attempt was made to select for significantly diverse genes (based on phylogenetic analysis) as these could possibly have varied functions as well. The various cis-acting elements identified, within the promoter regions of the genes examined, were the LRE (light-responsive element) and the stress-related elements like LTR (element involved in low-temperature responsiveness), MBS (MYB binding site involved in drought-inducibility), MRE (MYB binding site involved in light responsiveness) and the phytohormone related elements like ABRE (element involved in the abscisic acid responsiveness), ARE (part of an auxin-responsive element), SARE (element involved in salicylic acid responsiveness), GRE (gibberellin-responsive element) and JARE (element involved in the methyl jasmonate or MeJA responsiveness). The most abundant cis-acting element identified in all the promoter sequences was the LRE element followed by MBS and ABRE elements (Fig. 4).

A search for various, well-characterized, drought-responsive cis-acting elements across the promoters of RNA helicase genes resulted in identification of the elements with the core sequences ACGT (drought stress and aging response element), TAACTG (MYB2AT element) and A/CCCGAC (dehydration-responsive element).

### Physiochemical properties of the CaDEAD, CaDEAH and CaDExD/H-box proteins

The mean length of the CaDEAD-box proteins was 669 amino acids with CaDEAD5 being the largest (2381 amino acids) and CaDEAD24 being the smallest (108 amino acids). For the CaDEAH-box proteins, the length ranged from 691 amino acids (CaDEAH23) to 1739 amino acids for CaDEAH8, with an average of 1062 amino acids. Amongst the CaDExD/H*-*box proteins, the CaDExD/H62 protein was the smallest (182 amino acids) and the CaDExD/H54 was the largest, composed of 3496 amino acids. The mean length of these proteins was 1136 amino acids. The mean theoretical pI of all these proteins (CaDEAD, CaDEAH and CaDExD/H) was 7.44 (Supplementary Table S3). The vast majority of the chickpea RNA helicase proteins were predicted to be localized within the nucleus. A few were predicted to be localized in cellular organelles like chloroplast and mitochondrion. The latter might be responsible for regulation of organelle specific RNA metabolism.

### The structural features of the CaDEAD, CaDEAH and CaDExD/H-box proteins

**Figure 5:**
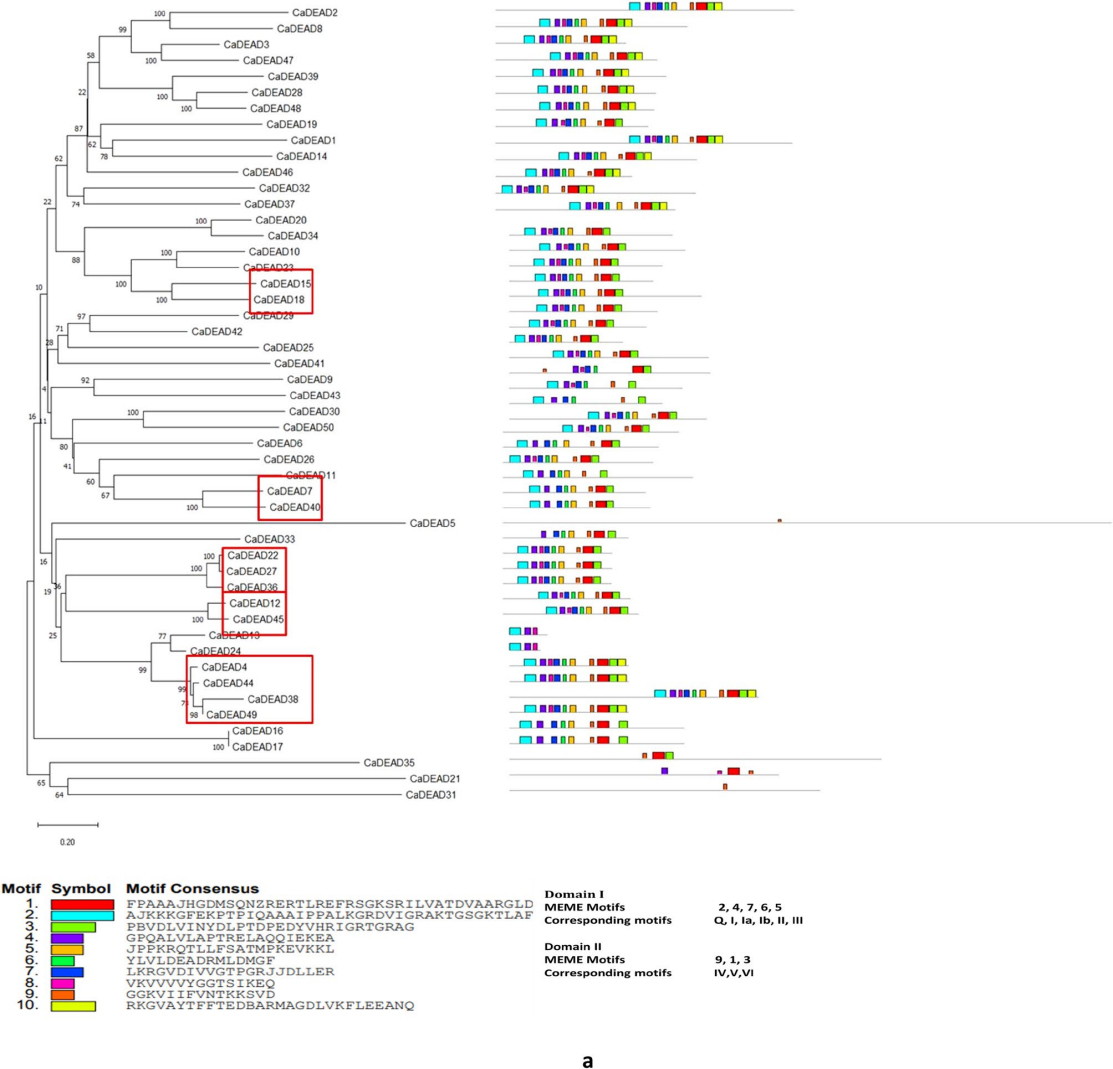

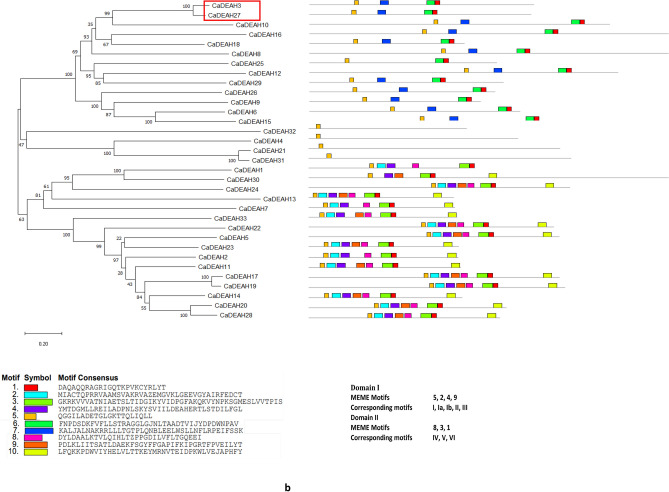

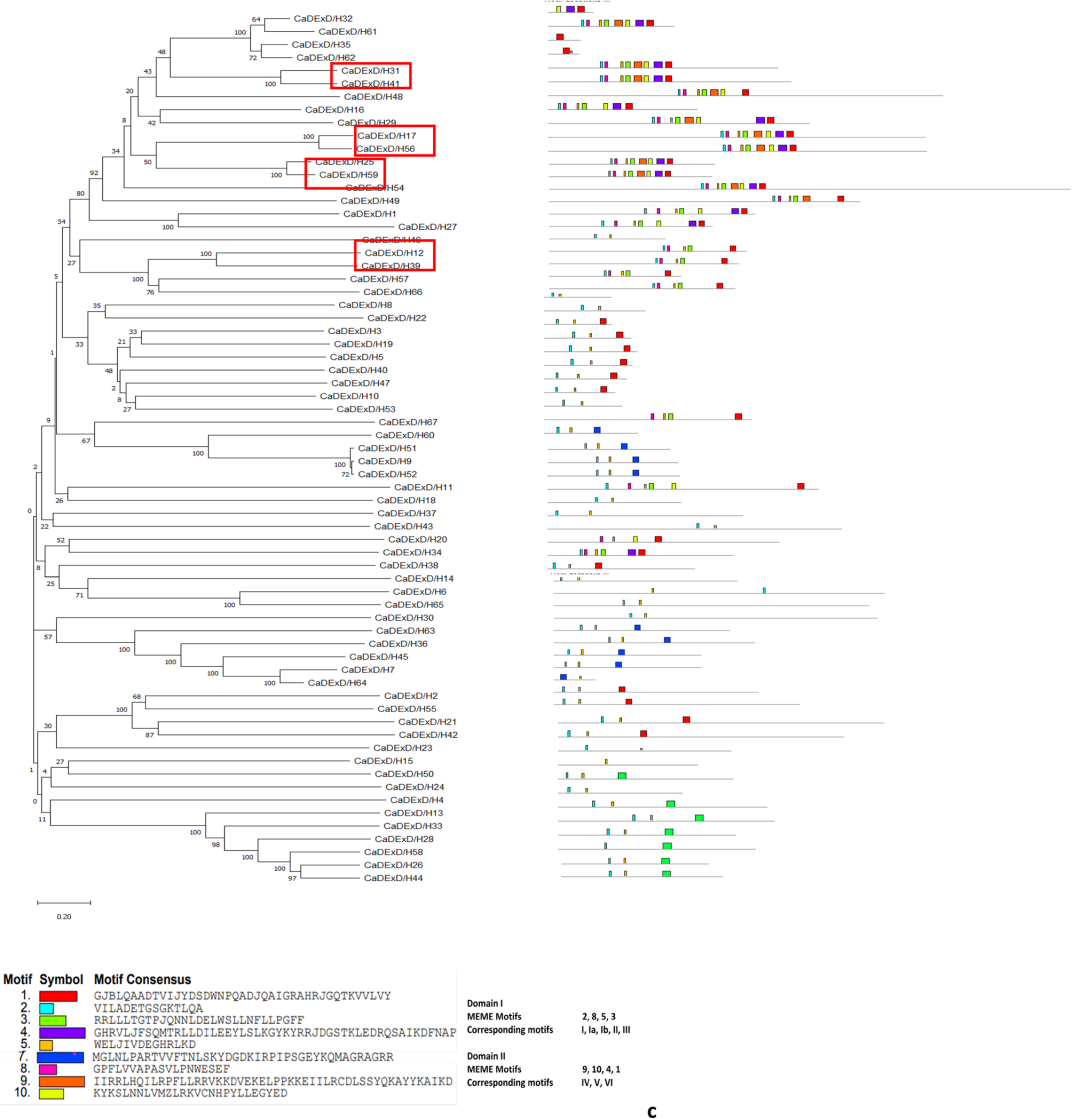
The distribution of conserved motifs in the identified RNA helicase proteins in chickpea. The horizontal coloured boxes indicate conserved motifs within each protein with description of the consensus sequences present at the motifs, described below the figures. Neighbor-joining tree of the proteins is shown on the left. Numbers at the nodes indicate bootstrap values. Duplicate gene pairs are enclosed in red colored boxes. (**a**) The CaDEAD-box helicases. (**b**) The CaDEAH-box helicases. (**c**) The CaDExD/H*-*box helicases.

The N-terminal sequence was highly variable compared to the C-terminal sequence for all the three classes of RNA helicase proteins. The nine characteristic motifs belonging to the two domains, domains I and II, could be identified in the majority (46/50) of DEAD-box RNA helicase proteins (Fig. 5a). The domain I consisted of MEME motifs 2, 4, 7, 6 and 5 while the domain II consisted of MEME motifs 9, 1 and 3. The MEME motif 2 consisted of motifs Q and I, MEME motifs 4, 7, 6 and 5 contained the characteristic motifs Ia, Ib, II (DEAD) and III, respectively. MEME Motif 8 was additionally found between the motifs 4 and 7 in some proteins. The MEME motifs 9, 1 and 3 correspond to the motifs IV, V and VI, respectively of the domain II. The additional motif 10 was found within the domain II of some of the DEAD-box proteins. Amongst the nine characteristic motifs, the highest degree of sequence conservation was observed for the motifs Q, I and II (~ 90%) while least sequence conservation was observed at motif IV (~ 60%) (Supplementary Fig. S6). The consensus sequence present at the motif I for the CaDEAD-box helicases was AKTGSGKT. This is a distinguishing feature between DNA and RNA helicases, as it has been reported that the DNA helicases (SF1) have GXXXXGKT (where X can be any amino acid, G: glycine; K: lysine; T: threonine), as a consensus sequence at the motif I whereas the RNA helicases have AXXXXGKT (A: alanine) as the consensus sequence at this motif^2^. It was observed that motif V (MEME motif 1) was present in all the CaDEAD proteins. The protein CaDEAD5 lacked most of the conserved sequence motifs but showed the presence of the conserved Helicase_C domain (pfam 00271, e-value = 1.49e−07) and was therefore considered to be a member of the CaDEAD-box proteins. The proteins CaDEAD21, CaDEAD31 and CaDEAD35 also lacked most of the characteristic sequence motifs of the DEAD-box proteins but shared a high degree of homology with the *Arabidopsis* DEAD-box proteins and were therefore considered as CaDEAD-box proteins.

Unlike the CaDEAD-box proteins, the CaDEAH-box proteins were found to be more variable in terms of motif sequence and organization. The Q motif was conspicuously absent in all the CaDEAH proteins and the motif II (DEAH) had a sequence conservation of ~ 50%. Motif I (MEME motif 5) was present in all the members of the CaDEAH subfamily (Fig. 5b). In addition to the conserved motifs which constitute the core helicase centre of the RNA helicase proteins, the CaDEAH-box proteins had additional conserved domains at the C terminus unlike the CaDEAD-box proteins. A few notable of these are the oligosaccharide-binding (OB)-fold domain (OB_NTP_bind, PF07717) almost always found in association with the HA2 domain (Helicase associated domain, PF04408). These domains interact strongly with the helicase core and this interaction is believed to be responsible for creating a less flexible helicase enzymatic activity than the DEAD-box helicases^34,35^.

The CaDExD/H *-*box proteins also lacked the Q motif for most of the members and the motif II had a sequence conservation of approx. 54%. Like the CaDEAH proteins, the motif I (MEME motif 2) could be identified in most of the CaDExD/H*-*box proteins. The other motifs were highly variable in composition as compared to the CaDEAD and CaDEAH-box proteins (Fig. 5c).

The CaDEAD-box proteins were the most conserved in terms of structure with an average pairwise distance of 1.38 as compared to the CaDEAH and CaDExD/H *-*box proteins which had an average pairwise distance of 2.0 and 2.27, respectively. It was also observed that the proteins encoded by the duplicated gene pairs (enclosed in red boxes in the dendrograms) within each subfamily, shared a very high degree of sequence homology (in terms of protein lengths, motif structure and organization), with high bootstrap support values (Fig. 5a–c).

**Figure 6:**
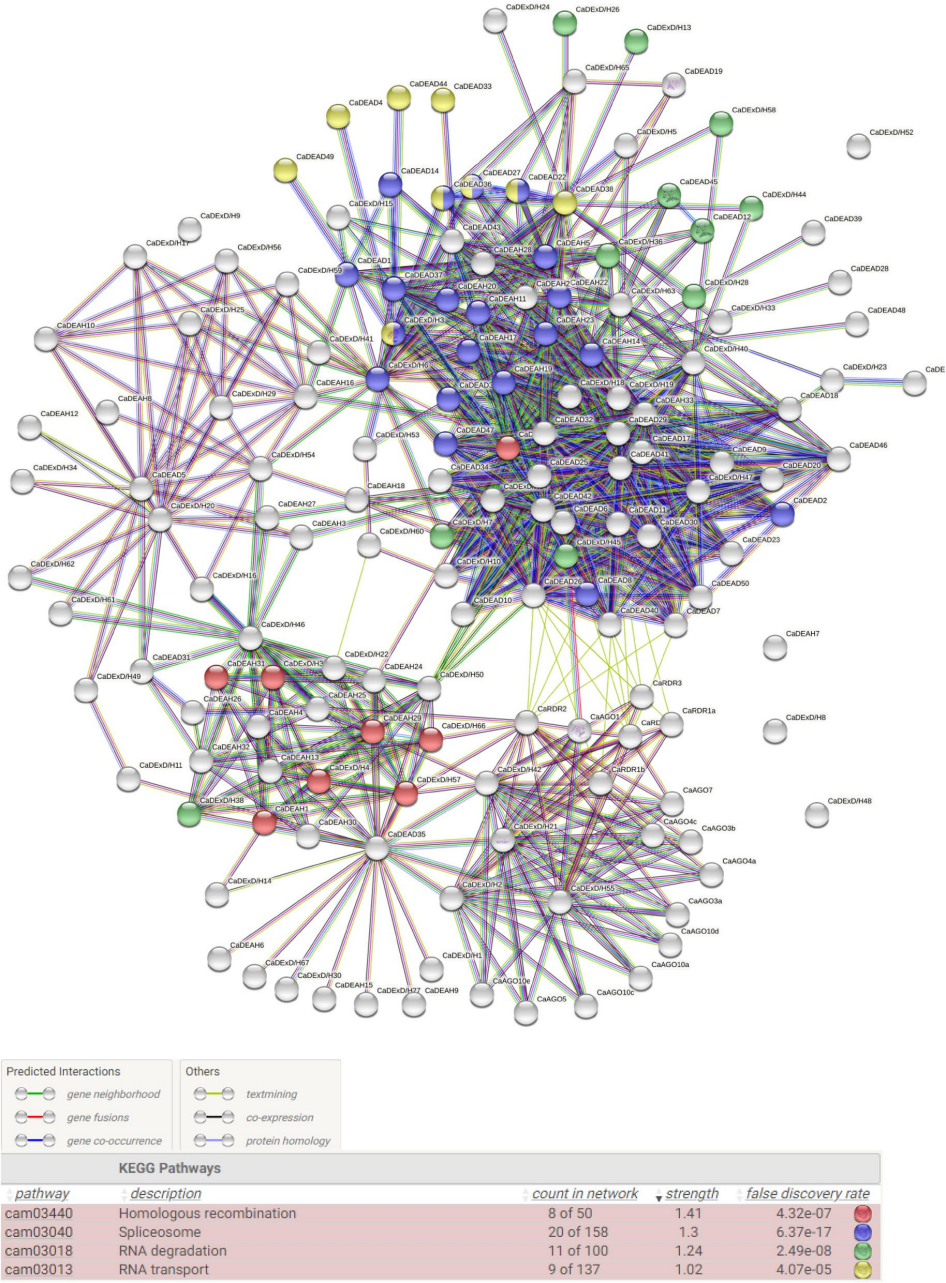
The protein–protein interaction networks for the chickpea RNA helicases (CaDEAD, CaDEAH and CaDExD/H*-*box proteins) and sRNA associated proteins (CaAGO and CaRDR). The experimentally validated interactions, as reported in previous published papers, are represented with pink edges. The filled nodes depict the proteins with known 3D structures. Colour of the nodes represent the KEGG pathway GO terms (www.kegg.jp/kegg/kegg1.html).

An interaction network consisting of 968 edges, with a PPI (protein–protein interaction) enrichment p value of < 1.0 × 10^–16^ was obtained on analysis of protein–protein interaction among the chickpea RNA helicases and sRNA processing enzymes (Fig. 6).The network consisted of only interactions with a minimum confidence score of 0.7 (high). The interacting proteins were predicted to be involved in KEGG pathways^36^ like homologous recombination, splicing, RNA degradation and transport (FDR < 10^–5^). An interactive module involving the proteins CaDEAD35, CaDExD/H45, CaDExD/H57 and CaDExD/H66, as major interacting partners with sRNA associated proteins (CaAGO, CaRDR) was also identified, suggestive of close association of these proteins in regulation of sRNA biogenesis and processing in chickpea. Within the network of sRNA associated proteins, we could also identify putative CaDCL (Dicer-like) proteins belonging to the CaDExD/H subfamily. The proteins CaDExD/H2, 21, 42 and 55 which interact with various CaAGO and CaRDR proteins are putative DCL proteins in chickpea.

### Phylogenetic relationship of the CaDEAD, CaDEAH and CaDExD/H-box proteins with RNA helicases of other plant species

**Figure 7:**
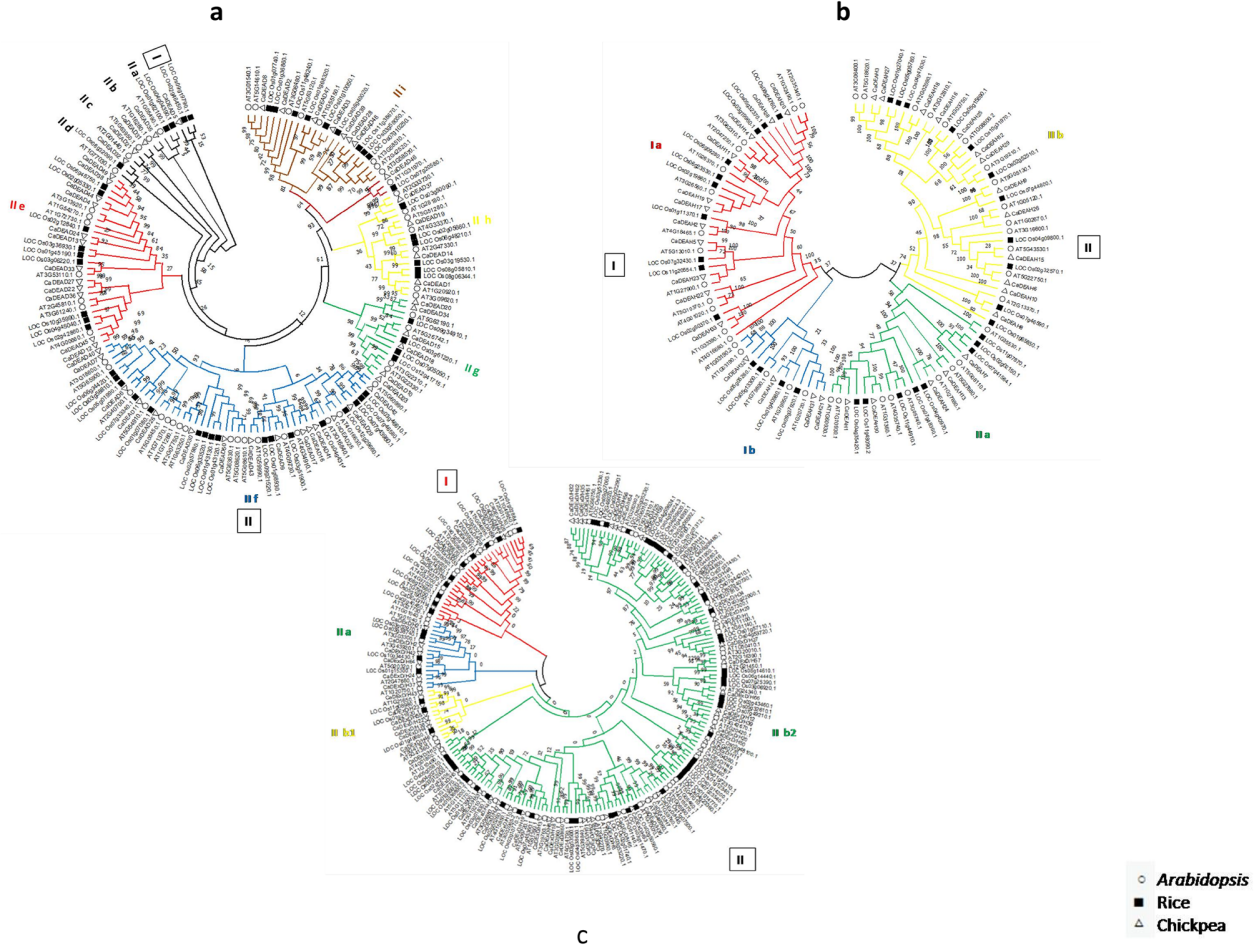
Phylogenetic relationships of the RNA helicases from *Arabidopsis thaliana*, *Cicer arietinum* and *Oryza sativa*. The trees were constructed by using the MEGA v 7 software (neighbour joining method with 1000 bootstrap replicates). Numbers at the nodes indicate bootstrap values. The coloured branches indicate the different clusters identified through the analysis. (**a**) DEAD-box helicases. (**b**) DEAH-box helicases. (**c**) DExD/H-box helicases.

In order to examine the evolutionary relationships amongst members of each of the three RNA helicase subfamilies, phylogenetic analysis with 151 DEAD-box proteins (50 *At*, 51 *Os* and 50 *Ca*), 106 DEAH-box proteins (40 *At*, 33 *Os* and 33 *Ca*) and 203 DExD/H-box (71 *At*, 65 *Os* and 67 *Ca*) proteins was performed by using the neighbour-joining method in MEGA 7.0. The DEAD-box proteins could be clustered into two major groups (Groups I and II). The group II could be further divided into nine subgroups (II a–II i). The average pairwise distance for these proteins was estimated to be 1.41, with the proteins LOC_Os01g45190.1 and LOC_Os03g36930.1 with least pairwise distance of 0 and the proteins AT1G16280.1 and CaDEAD31 with a pairwise distance of 2.79. CaDEAD33 could be identified as a homolog of the *Arabidopsis* LOS4 protein (AT3G53110) (Fig. 7a). The DEAH proteins belonging to the three species could be broadly categorized into two groups (Groups I and II), each of which could be further divided into two subgroups (Fig. 7b). Similarly, the DExD/H*-*box proteins were clustered into two broad groups Groups I and II. The Group II was further divided into two smaller subgroups (II a and II b). The subgroup II b was further clustered into a smaller subgroup II b1 and a larger subgroup II b2 (Fig. 7c). Higher sequence diversity was observed for the DEAH and DExD/H *-*box proteins with an average pairwise distance of 2.02 and 2.39, respectively. For the proteins CaDExD/H35 and AT3G11200, the highest pairwise distance of 3.28 was observed. CaDExD/H36 was identified as a potential homolog of the AtHELPs protein (AT3G46960).

### Digital expression profiles of the RNA helicase gene family in chickpea

**Figure 8:**
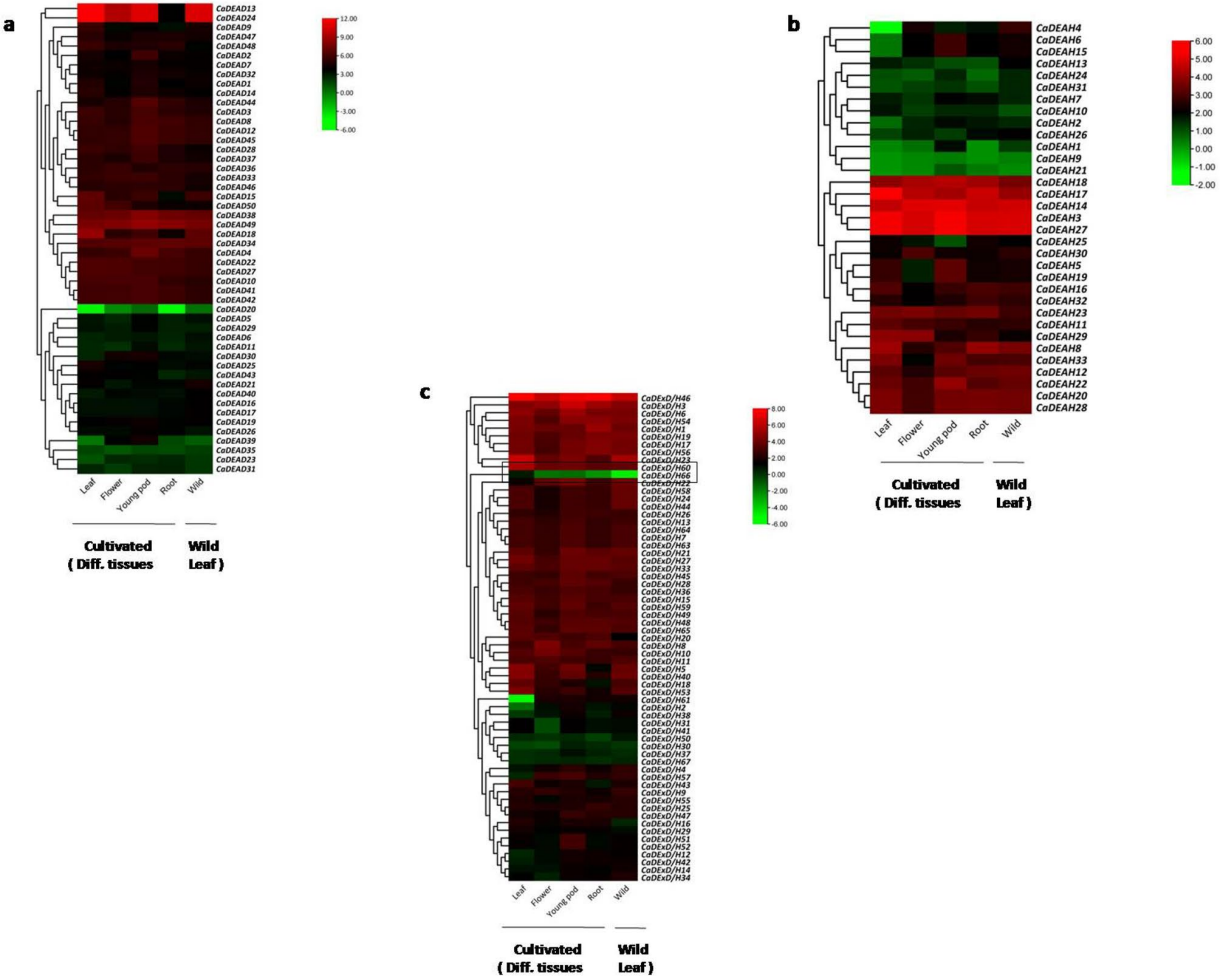
The gene expression patterns of the RNA helicase genes in chickpea. The FPKM values obtained from RNA-Seq data for different tissues (leaves, roots, flowers and young pod) of the genotype, ICC 4958 (cultivated, *C. arietinum*) and leaves of wild chickpea accession, PI 489777 (*C. reticulatum*) were log 2 transformed for the *CaDEAD* (**a**), *CaDEAH* (**b**) and *CaDExD/H-*box (**c**) genes and used for generation of heatmaps. The heatmaps were generated using TBtools Version 1.098693 (https://github.com/CJ-Chen/TBtools/releases).

The expression patterns for most of the RNA helicase genes was conserved across all the different tissues examined (Fig. 8). However, for a few genes, tissue specific expression was observed. Both the genes *CaDEAH4* and *CaDExD/H61* (Fig. 8b,c) were substantially down-regulated in the leaf tissues of the cultivated genotype as compared to the other tissues or the leaf tissues of the wild genotype. These genes might play different roles in the leaf tissues of the wild and cultivated species. The gene *CaDExD/H66* was down-regulated in the leaf tissues of the wild genotype relative to all the tissues of the cultivated genotype. This gene (LOC101491973) is a putative *CLSY* gene (CLSY family), whose homolog in *Arabidopsis* encodes for a protein involved in regulation of RNA Polymerase. IV, in turn controlling the level of DNA methylation both at the global and at the locus-specific level^37^.

**Figure 9:**
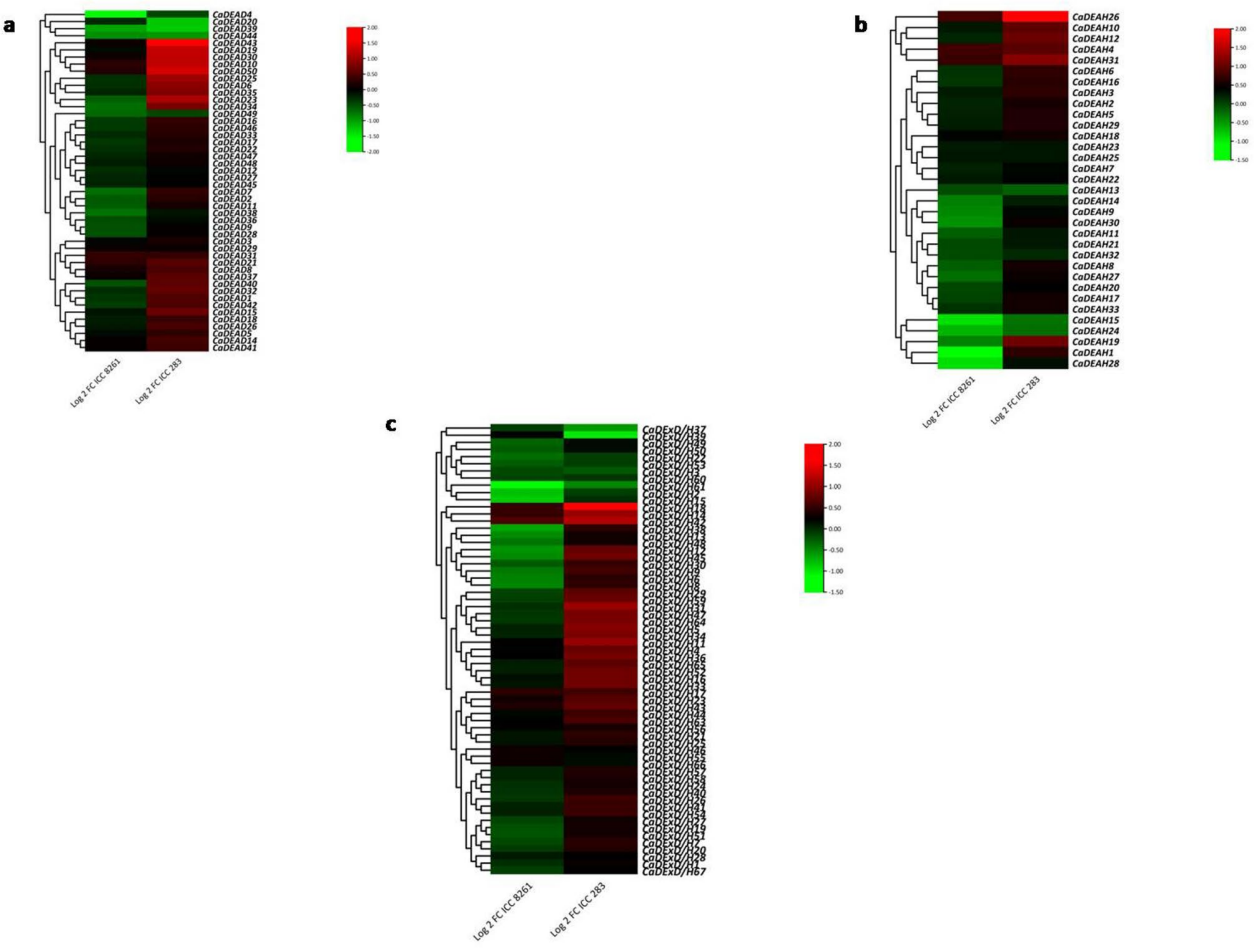
The heatmaps depicting the log2 FC for the gene expression levels of the *CaDEAD* (**a**)*, CaDEAH* (**b**) and *CaDExD/H-*box (**c**) genes in response to drought stress conditions. These values were obtained from the publicly available transcriptome data for the leaf tissues of two genotypes contrasting for drought stress tolerance; ICC 8261 (drought tolerant) and ICC 283 (drought sensitive). The heatmaps were generated using TBtools Version 1.098693 (https://github.com/CJ-Chen/TBtools/releases).

To examine the drought-responsive expression of the chickpea RNA helicase gene family the log2FC values obtained from the RNA-Seq data derived from the leaf tissues of two genotypes, exposed to drought stress and collected at the shoot apical meristem formation stage, ICC 8261 (DT) and ICC 283 (DS), were analysed. It was observed that for the *CaDEAD* and *CaDExD/H-*box subfamilies, a considerably higher number of genes were down-regulated in response to drought stress for the DT genotype as compared to the DS genotype (Fig. 9). The genes *CaDEAD46* and *CaDEAD50*, which are homologs for *AtSTRS1* (At1g31970) and *AtSTRS2* (At5g08620) genes (Stress Response Suppressor genes, negative regulators of stress-responsive TFs) showed different expression profiles in the two genotypes (Fig. 9a). Both the genes were relatively up-regulated in the DS genotype compared to the DT genotype.

### Morpho-physiological attributes of chickpea genotypes in response to drought stress treatment

**Figure 10:**
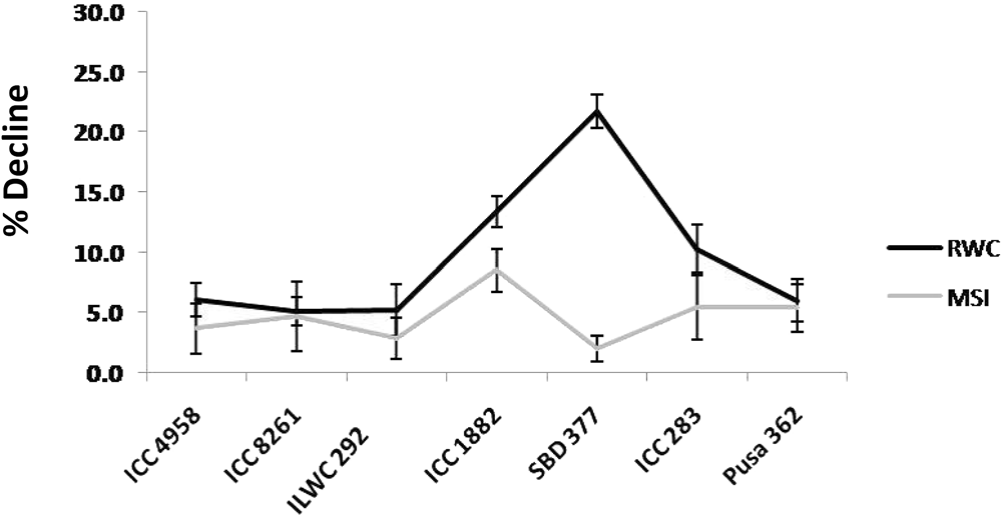
The percentage (%) reduction in leaf relative water content (RWC) and membrane stability index (MSI) of the drought stress treated plants (T24) over the control (C24) or well-watered plants for seven different genotypes, namely ICC 4958, ICC 8261, ILWC 292, ICC 1882, SBD 377, ICC 283 and Pusa 362. ILWC 292 belongs to the wild species *C. reticulatum*, while the remaining six genotypes belong to the cultivated chickpea species, *C. arietinum*. ICC 4958, ICC 8261, ILWC 292, Pusa 362 are drought tolerant and ICC 1882, SBD 377 and ICC 283 are drought sensitive. Data for RWC and MSI were recorded for the C24 and T24 plants on 117 DAS. An average value of five biological replicates/genotype/treatment (n = 5) were used for both the traits, to plot the line graphs. Vertical lines represent the mean ± SE.

On day 0 (onset of drought stress treatment) i.e., 93 DAS, there was no significant difference in RWC of the plants across all the seven genotypes. However, on day 12 after drought stress initiation (105 DAS) there was a significant reduction (p < 0.05) in RWC of the T12 plants compared to the C12 plants for as many as five genotypes. The gap between RWC of the C12 and T12 plants was observed to be least and highest for ICC 4958 and ICC 283, respectively (Supplementary Fig. S7a). On day 24 of drought stress (117 DAS), at a stage when water was withheld for 24 days from the drought stressed plants (T24), the highest significant (p < 0.05) reduction in RWC of the T24 over the C24 plants was observed for the genotype SBD 377 (21.7%) (Fig. 10). However, it is noteworthy that the C24 plants of SBD 377 were able to maintain a higher RWC (75%) on day 24 as compared to the C24 plants of other genotypes. This resulted in widening of the gap/difference between the C24 and T24 plants for SBD 377 compared to other genotypes. On the contrary, ICC 283, showed highest RWC difference between the C12 and T12 plants, but this gap decreased on day 24 as the RWC of the C24 plants also declined at this stage.

For MSI on day 24, highest reduction for the T24 plants over the C24 plants, was observed for the genotype ICC 1882 (8.5%) while for the genotype SBD 377 least reduction (2%) was observed (Fig. 10, Supplementary Fig. S7b).

Based on both the parameters, least reduction in T24 plants over the C24 plants for both RWC and MSI, 5.2 and 2.9%, respectively (p < 0.05) was observed for the genotype ILWC 292 followed by ICC 8261 (5.1 and 4.7%) and ICC 4958 (6.1 and 3.7%). Highest reduction for both the parameters was observed for the T24 plants of the genotype ICC 1882 (13.4 and 8.5%), followed by ICC 283. For, SBD 377 it was observed that the % age reduction in RWC and MSI was not collinear. While the SBD 377 T24 plants exhibited a very high reduction in RWC over the C24 plants (21.7%), the percentage reduction in MSI (2%) was very less for the same set of plants (Fig. 10).

**Figure 11:**
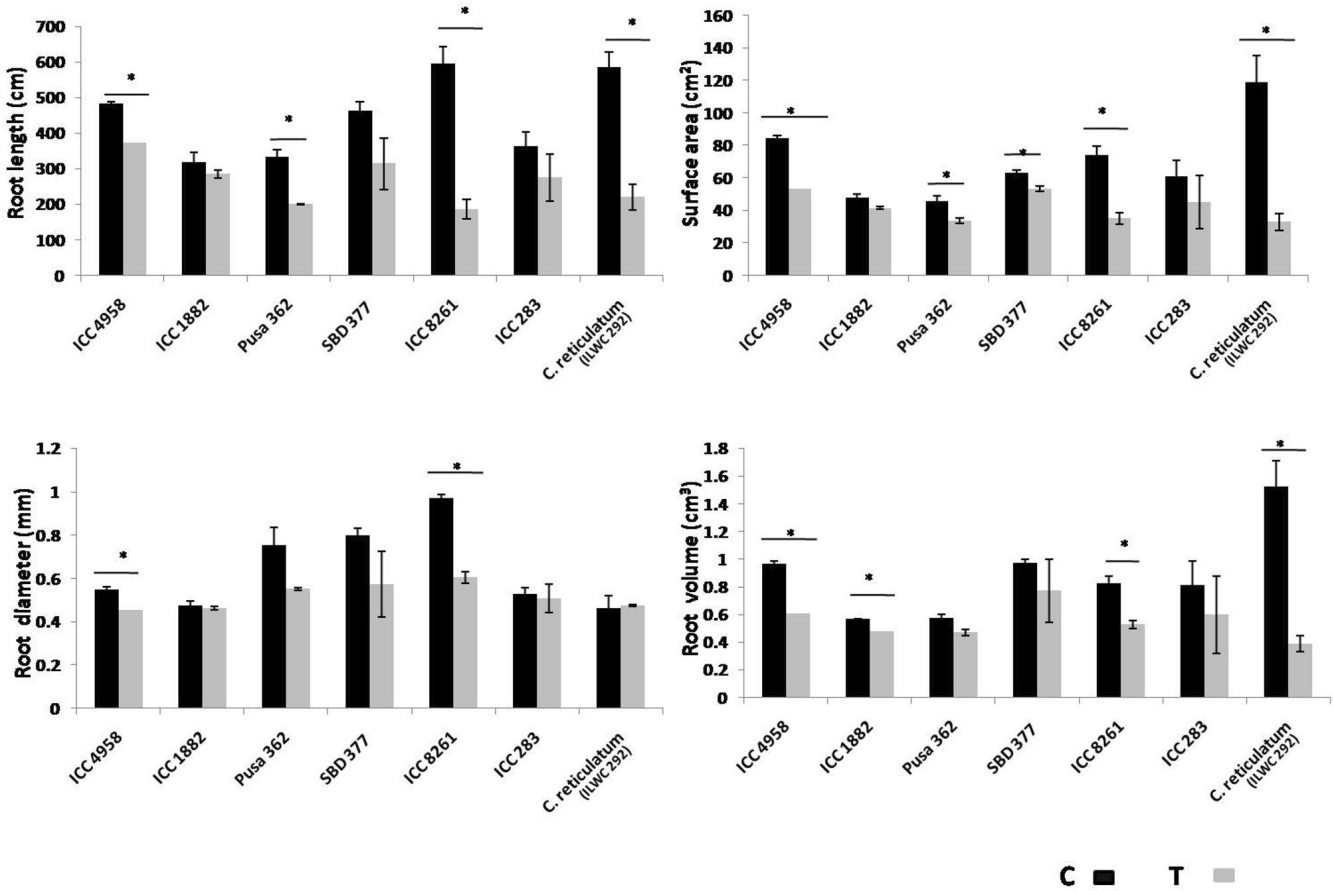
Differences in root morphological traits (root length, surface area, root diameter and root volume) between the control, well-watered (C24) and drought stress treated (T24) plants, for the seven genotypes used in the study. The data for the traits was recorded on 117 DAS. ILWC 292 belongs to the wild species *C. reticulatum*, while the remaining six genotypes belong to the cultivated chickpea species, *C. arietinum*. ICC 4958, ICC 8261, ILWC 292, Pusa 362 are drought tolerant and ICC 1882, SBD 377 and ICC 283 are drought sensitive. Error bars indicate standard errors of means of three biological replicates/genotype/treatment (n = 3). Statistical significance of differences in means, was tested by Student’s t-test. The *symbol above horizontal lines (C and T) represent significant difference at p < 0.05.

Data for the root morphological traits were recorded for the C24 and T24 plants for all the seven genotypes. The mean RL was highest for the genotypes ICC 8261 (595 cm) and ILWC 292 (583 cm) and least for ICC 1882 (317 cm). The highest significant reduction (p < 0.05) between the C24 and T24 plants was also observed for the two genotypes, ICC 8261 and ILWC 292. For ICC 4958 there was least significant reduction in RL between the C24 and T24 plants (p < 0.05). Compared to all the other traits like RL, SA and RV, the reduction in RD of the T24 plants when compared to the C24 plants, was much lesser (Fig. 11).

### Drought-responsive RNA helicase genes in chickpea identified through qRT-PCR

**Figure 12:**
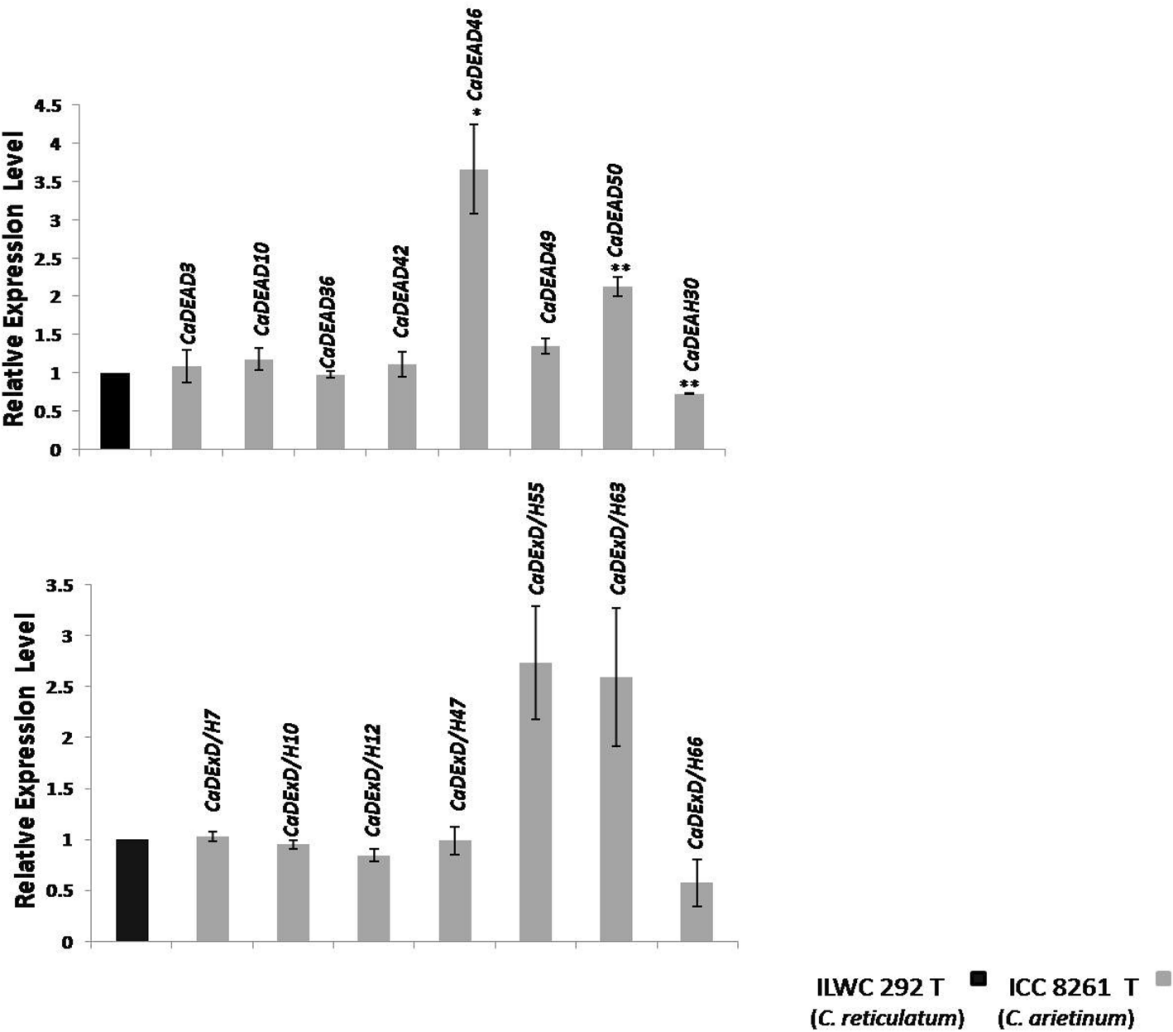
The relative expression levels of selected 15 RNA helicase genes (7 *CaDEAD*, 1 *CaDEAH* and 7 *CaDExD/H*-box genes) in leaf tissues of two genotypes, ILWC 292 (*C. reticulatum*) and ICC 8261 (*C. arietinum*), in response to drought stress (T24), as estimated by qRT-PCR. The T24 treatment indicates water being withheld for 24 days beginning with 93 DAS. The relative expression levels which were normalized to *GAPDH* were determined by the comparative CT method (2 − ΔΔCT). The expression level of genes for ILWC 292 T24 was considered as control (expression = 1). Three biological and technical replicates were used for each experiment. Error bars indicate standard errors of means. The *symbol above bars indicates significant difference at p < 0.05 and **indicates significant difference at p < 0.01.

Based on transcript abundance, we could identify the gene *CaDEAD50* as a drought-responsive RNA helicase gene in chickpea. On exposure to drought stress treatment, it was found to be up-regulated in ICC 8261 as compared to ILWC 292 (p value < 0.01) (Fig. 12). It was observed that the gene was up-regulated in both the genotypes on exposure to drought stress but the magnitude of up-regulation was higher in ICC 8261 as compared to ILWC 292 (Supplementary Fig. S8b,c). For the gene *CaDExD/H66* an opposite pattern was observed, on exposure to drought stress*.* While its expression did not change in ICC 8261 under drought stress, it was over-expressed in ILWC 292 on drought stress treatment. Therefore, its relative expression in ICC 8261 was lesser compared to ILWC 292 due to drought stress (Fig. 12). It is noteworthy that the proteins encoded by homologs of both these genes in *Arabidopsis* have been identified to play key roles in epigenetic regulation of gene expression, primarily by controlling the de novo methylation pathway^37,38^.

## Discussion

The cellular repertoire of different RNA species in eukaryotic organisms, has expanded immensely in the last decade or so. With the identification of non-coding RNAs like long non-coding RNAs (lncRNAs), small non-coding RNAs like miRNAS (microRNAs) and siRNAs (small interfering RNA) and other forms of non-coding RNA species in plants^39^ and with the continuing research to decipher their molecular functions, the enzymes which regulate their synthesis and metabolism, become important subjects of scientific research and investigation. The SF2 RNA helicases which consist of the DEAD, DEAH and DExD/H-box subfamilies, have been identified in several plant species^40^. Amongst the leguminous crops, this gene family has been identified in soybean (*Glycine max*)^4^. In the present study, we identified a total of 150 RNA helicase genes (50 *DEAD*, 33 *DEAH* and 67 *DExD/H*-box genes) across the chickpea genome. This number is comparable to the number identified in rice (149) but lesser than those in soybean (213) and *Arabidopsis* (161)^4^. The higher number of RNA helicase genes existing in soybean is expected owing to the larger genome size (1115 Mb) compared to that of chickpea (738 Mb). Also, since in soybean, genome duplication has occurred twice, which has led to the duplication of about 75% of soybean genes, the number of members identified for most of the gene families in soybean is always higher than in chickpea^41–43^. A plausible explanation for higher number of these genes identified in *Arabidopsis* as compared to chickpea, could be because of the differences in the genome assembly and its annotation. While for *Arabidopsis* assembly version TAIR10.1, 119 Mb of 125 Mb has been sequenced and annotated (TAIR10.1, GCF_000001735.4), for the chickpea assembly version GCF_000331145.1, 530 Mb of 738 Mb has been sequenced and annotated (NCBI Assembly database). The higher degree of genome characterization in *Arabidopsis* compared to chickpea could have led to the increased number of RNA helicase genes being identified in the former. It cannot be overruled that with higher number of gene annotations and protein coding genes being identified for chickpea in future, the number of genes reported for not only the RNA helicase gene family but for other gene families also, may increase. Inspite of the differences observed in the numbers of RNA helicase genes across the different species, the overall size in terms of % of genes is similar. In chickpea it is 0.45%, while it is 0.39% and 0.32% in rice and soybean, respectively^4^. The positions, lengths and number of introns within the chickpea RNA helicase genes was found to be highly variable for all the three subfamilies, as has been observed in *Arabidopsis*^15^. The gene duplication analysis revealed the presence of 15 pairs of paralogous genes in chickpea genome. There was a predominance of segmental duplication events which led to the expansion of the RNA helicase gene family in chickpea. It was observed that except for one pair *CaDExD/H12/CaDExD/H39* (protein identity of 57.5%), the gene expression levels of the genes belonging to a duplicated gene pair were similar across two different genotypes, which contrast for drought tolerance. The different expression profiles of these two genes is suggestive of their functional non-redundancy. It could be possible that the two genes acquired different functions after duplication (neofunctionalization). The promoter sequences of the helicase genes were enriched in light and drought-responsive elements. The light-responsive nature of the promoters could indicate towards a DNA/RNA-damage repair function of these helicases as it has been found that many of the helicases are induced in response to and to rectify the oxidative damage caused by UV-B exposure to nucleic acids^44^. The most abundant, drought-responsive element that was identified in as many as 13/20 genes examined was the drought stress and aging response element (ACGTATERD1). This element with ACGT as a core sequence has been previously reported in promoters of stress-responsive genes which are induced by ABA^45^.

Compared to the CaDEAD-box proteins, the CaDEAH and the CaDExD-box proteins, were larger in sizes and less conserved in terms of motif structures and organization. This observation is in agreement with previous reports in *Arabidopsis*, sweet potato, cotton, etc^4,12,13^. The diversity in terms of protein structures might confer functional diversity to the RNA helicases in chickpea.

The RWC and MSI are two of the most widely utilized traits to estimate the drought tolerance behaviour of different genotypes across several different crop species^46,47^, including chickpea^48^. Amongst the seven genotypes utilized in the present study, which exhibit differential drought tolerance, it was observed that the average % reduction for both RWC and MSI for the T24 plants over the C24 plants was lesser for the DT genotypes (5.5 and 3.8%, respectively) compared to the DS genotypes (15.1 and 5.3%, respectively). The genotypes ILWC 292 and ICC 8261 exhibited highest drought tolerance, based on least reduction for both RWC and MSI for the T24 plants vis-à-vis C24 plants. The superior performance of these genotypes under drought stress could be partly attributed to their relative late maturity compared to other genotypes used in the study. Owing to their longer maturity durations, these genotypes might have access to more water during the major part of their early growth. While for most genotypes there was a proportionate, collinear decline in RWC and MSI for the T24 plants over the C24 plants, for SBD 377 a non-collinear relationship was observed. While RWC reduction was highest (21.7%) for the T24 plants over the C24 plants, the reduction in MSI (2%) was least. Similar observations with the genotype have been made previously where RWC and MSI reduction of 21.6% and 9.9%, respectively, was reported on exposure to salinity stress^49^. In the same study, for the rest of the genotypes which included ICC 1882, ICC 4958, Pusa 362, etc., a much smaller difference was reported between the two trait values. Despite these differences, SBD 377 has conventionally been considered as a sensitive variety with respect to drought^50^ and moderately tolerant variety with respect to heat stress^51^. For the root traits, higher mean reduction for the T24 plants, for all the traits was observed for the DT genotypes compared with DS genotypes. Least mean reduction was observed for the trait RD. It has been observed that under low soil moisture conditions, the roots demonstrate agravitropic growth response, during which the root length decreases but the diameter increases. This behaviour of roots has been previously reported in chickpea in response to drought and salinity stress^52,53^.

The gene expression analysis through qRT-PCR, was performed to identify drought-responsive RNA helicase genes in chickpea. The *CaDEAD50* gene, was up-regulated in the cultivated variety relative to the wild genotype on drought stress exposure. The gene *CaDExD/H66,* on the other hand was down-regulated in the cultivated variety when compared to the wild genotype, under drought stress. The proteins encoded by the *CaDEAD50* and *CaDExD/H66* genes, share a high degree of homology with the *Arabidopsis* STRS2 protein (NP_196479, 74.5% identity, query coverage of 83%, e-value = 0) and CLSY protein (NP_172040, 49.6% identity, query coverage of 57%, e-value = 0), respectively. Both these proteins are known to be involved in the RdDM pathway and bring about differential methylation at specific loci, the stress-responsive genes^54^ by the STRS proteins and both at specific loci (siRNA loci) and across the genome by the CLSY proteins^37^. In fact we were able to identify five RNA helicase genes, i.e., the *CaDEAD46*, *CaDEAD50*, *CaDExD/H12*, *CaDExD/H39* and *CaDExD/H66*, as potential homologs of the *Arabidopsis STRS1*, *STRS2*, *CLSY1*, *CLSY2* and *CLSY3* genes, respectively. The proteins encoded by these *Arabidopsis* genes have been shown to control DNA methylation (mC) through the RdDM pathway (involving the various RNA Polymerase enzymes). The transcriptome data (Fig. 9) shows that while some genes like *CaDEAD46* and *CaDExD/H66* show little change in expression in the DT or DS variety in response to drought stress, the genes *CaDEAD50*, *CaDExD/H12* and *CaDExD/H39* were all differentially expressed amongst the DT and DS varieties, on exposure to drought stress. However, based on the qRT-PCR results, since we could identify the genes *CaDEAD50* and *CaDExD/H66* as drought-responsive and also keeping in view that the CaDExD/H66 protein was found to interact (confidence score > 0.7) with the CaRDR2 protein, (protein–protein interaction network analysis), we contemplate the role of epigenetic regulation of gene expression in response to drought stress in chickpea, mediated by the CaDExD/H66 protein. In a study which described the organ-specific DNA methylation patterns in chickpea, it was observed that there was a substantially higher number of differentially methylated regions (DMRs) (23,265), identified for the leaf tissues collected from the wild chickpea (*C. reticulatum*) genotype PI 489777 when compared to that of cultivated chickpea^26^. Amongst these the hypomethylated DMRs constituted the bigger fraction than the hypermethylated DMRs. The *CaDExD/H66* gene was also found to be up-regulated in the leaf tissues of the cultivated variety of chickpea, relative to the leaf tissues from the wild species, under control conditions (the transcriptome data generated in the same study, from different tissues of wild and cultivated species, Fig. 8c and corroborated by the qRT-PCR analysis, Supplementary Fig. S8a, present study). In *Arabidopsis,* in the *clsy* mutants, a high degree of overlap was observed between hypomethylated DMRs and reduced 24nt-siRNA clusters, especially in the non-CG contexts^37^. Based on all these observations, it can be hypothesized that the gene *CaDExD/H66*, could possibly be involved in regulation of DNA methylation levels in chickpea by regulating siRNA production, in conjunction with other proteins like the RDRs, AGO and DCL proteins.

## Conclusion

This study provides a detailed and comprehensive analysis of the RNA helicases of the SF2 superfamily in chickpea. A total of 150 RNA helicase genes belonging to the DEAD, DEAH and DExD/H-box subfamilies were identified across the chickpea genome. The CaDEAD-box proteins were found to be most conserved in terms of protein structure amongst all the three subfamilies. Also, highest number of duplicated gene pairs were identified for the *CaDEAD*-box genes as compared to the *CaDEAH* and *CaDExD/H*-box genes. Gene expression analysis led to the identification of drought-responsive RNA helicase genes in chickpea. The gene *CaDExD/H66* was identified as a drought-responsive helicase gene, which could potentially be involved in regulation of DNA methylation patterns in chickpea through the RdDM pathway.

## Supplementary Information

Supplementary Figures.

Supplementary Tables.

## Data Availability

Two publicly available data sets were utilized for digital gene expression analysis. The first comprised of the FPKM values from RNA-seq. data retrieved from NCBI GEO dataset, BioProject ID: PRJNA401922. The second dataset corresponded to the log 2 FC values of gene expression derived from the RNA-Seq dataset, BioProject ID: PRJNA413294.
