# Supplementary material for: Genome-wide identification, in silico characterization and expression analysis of the RNA helicase gene family in chickpea (C. arietinum L.)
Source: Sci Rep. 2022 Jun 13;12:9778. doi: 10.1038/s41598-022-13823-9 (PMC9192698; doi:10.1038/s41598-022-13823-9)
Supplement: Supplementary file 1 — Supplementary Figures. [file 41598_2022_13823_MOESM1_ESM.docx]

**Genome-wide identification, *in silico* characterization and expression analysis of the RNA helicase gene family in chickpea (*C. arietinum* L*.*)**

**Sheel Yadav^1^, Yashwant K. Yadava^1^, Deshika Kohli^1^, Shashi Meena^2^, Gopal Kalwan^1^, C. Bharadwaj^3^, Kishor Gaikwad^1^, Ajay Arora^2^ & P.K. Jain^1*^**

^1^ICAR-National Institute for Plant Biotechnology, New Delhi, 110012, India

^2^Division of Plant Physiology, ICAR-Indian Agricultural Research Institute, New Delhi, 110012, India

^3^Division of Genetics, ICAR-Indian Agricultural Research Institute, New Delhi, 110012, India

***Corresponding author e - mail** : [jainpmb@gmail.com](mailto:jainpmb@gmail.com)

**ORCID ID (P.K. Jain) :** https://orcid.org/0000-0001-5199-4429


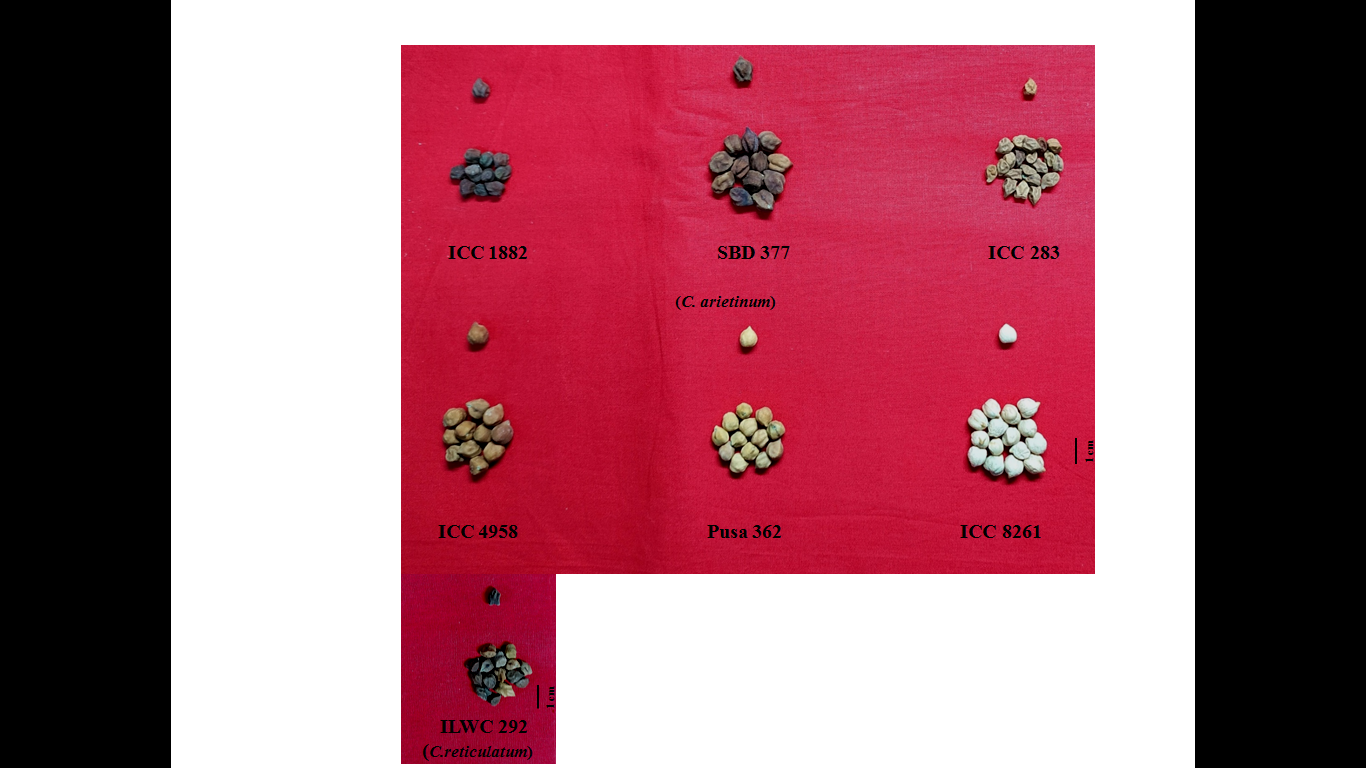


**Supplementary Figure S1.** Seed morphologies of the seven different genotypes of chickpea used in the present study. The genotypes ICC 1882 (desi), SBD 377 (desi), ICC 283 (desi), ICC 4958 (desi), Pusa 362 (desi) and ICC 8261 (kabuli) belong to the cultivated chickpea species (*C. arietinum*); the genotype ILWC 292 belongs to the wild species (*C. reticulatum*). ICC 4958, ICC 8261, ILWC 292, Pusa 362 are drought tolerant (DT) and ICC 1882, SBD 377 and ICC 283 are drought sensitive (DS).


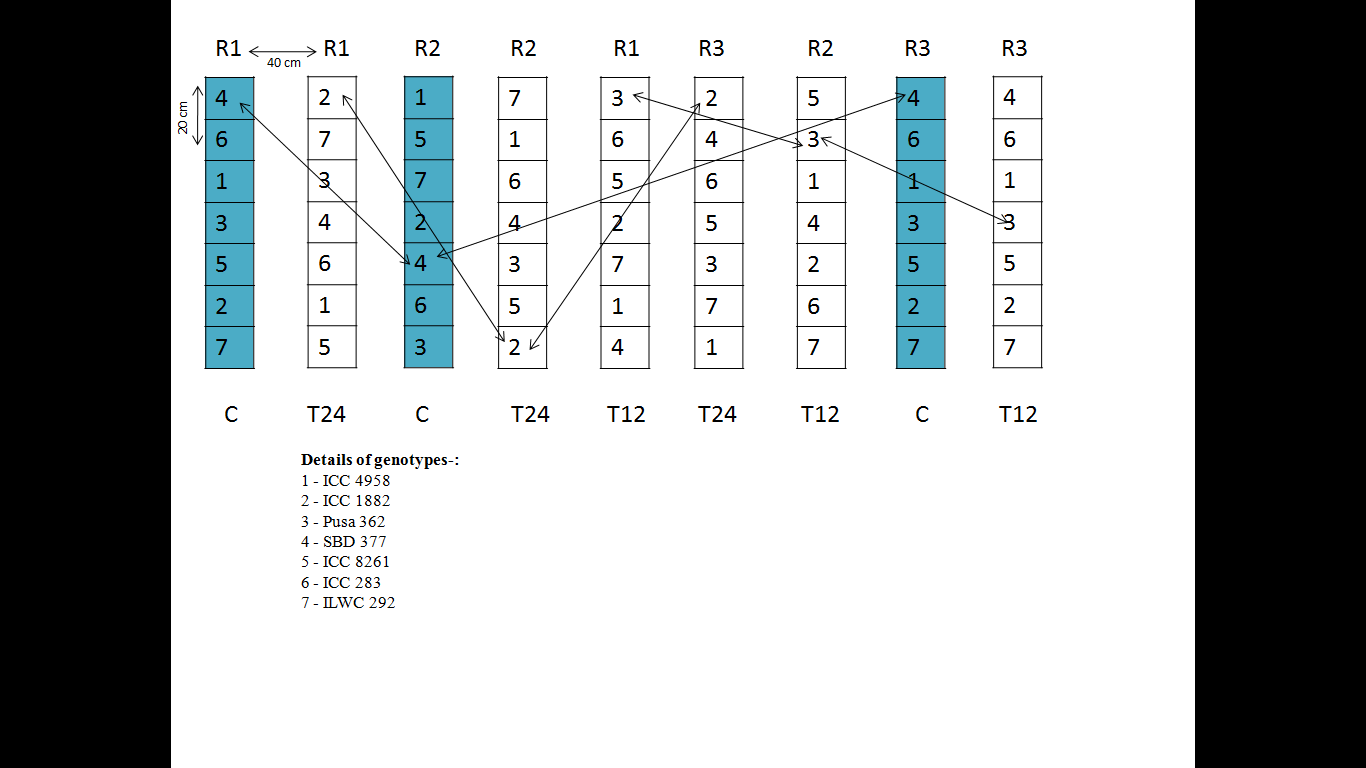


**Supplementary Figure S2.** Diagram representing the experimental layout depicting the arrangement of bags. 1,2,3,4,5,6 and 7 are the numbers given to the seven different genotypes used in the study; C (blue in colour): well watered (WW), control (C) plants for all the seven genotypes; T12 and T24: drought stress treated plants (T) with water being withheld for 12 (T12) and 24 (T24) days, beginning at 93 DAS, which is also referred to as day 0 since it was the day of drought stress initiation for the drought stressed plants; arrows are drawn to show the three biological replicates (R1,R2 and R3) of a genotype for the three different treatments (C, T12 and T24). Day 0 of stress implies 93 DAS; day 12 is 105 DAS and day 24 is also 117 DAS.


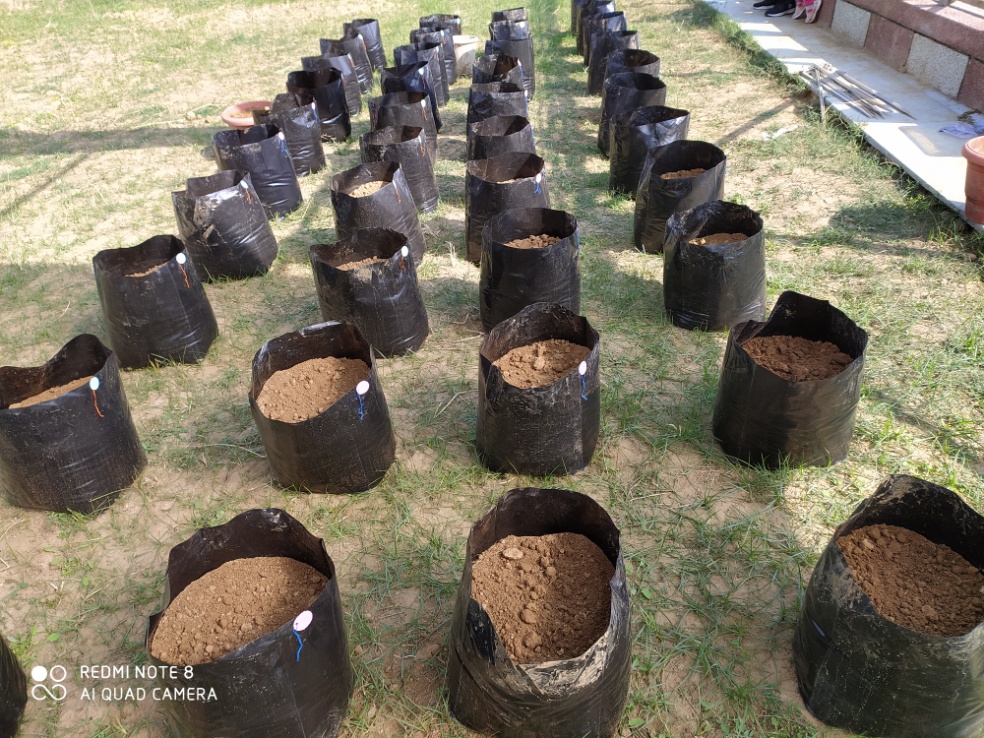

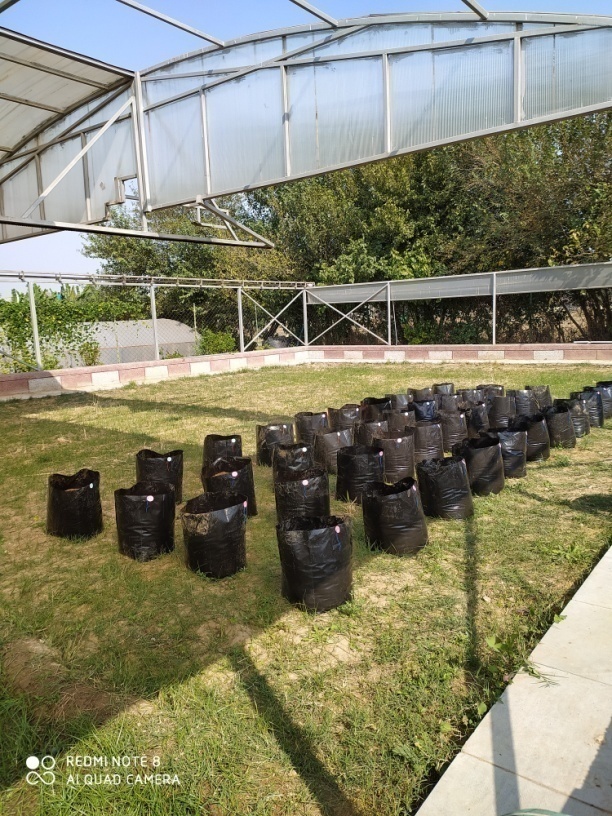


**a**


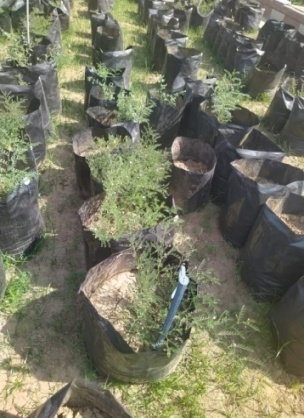

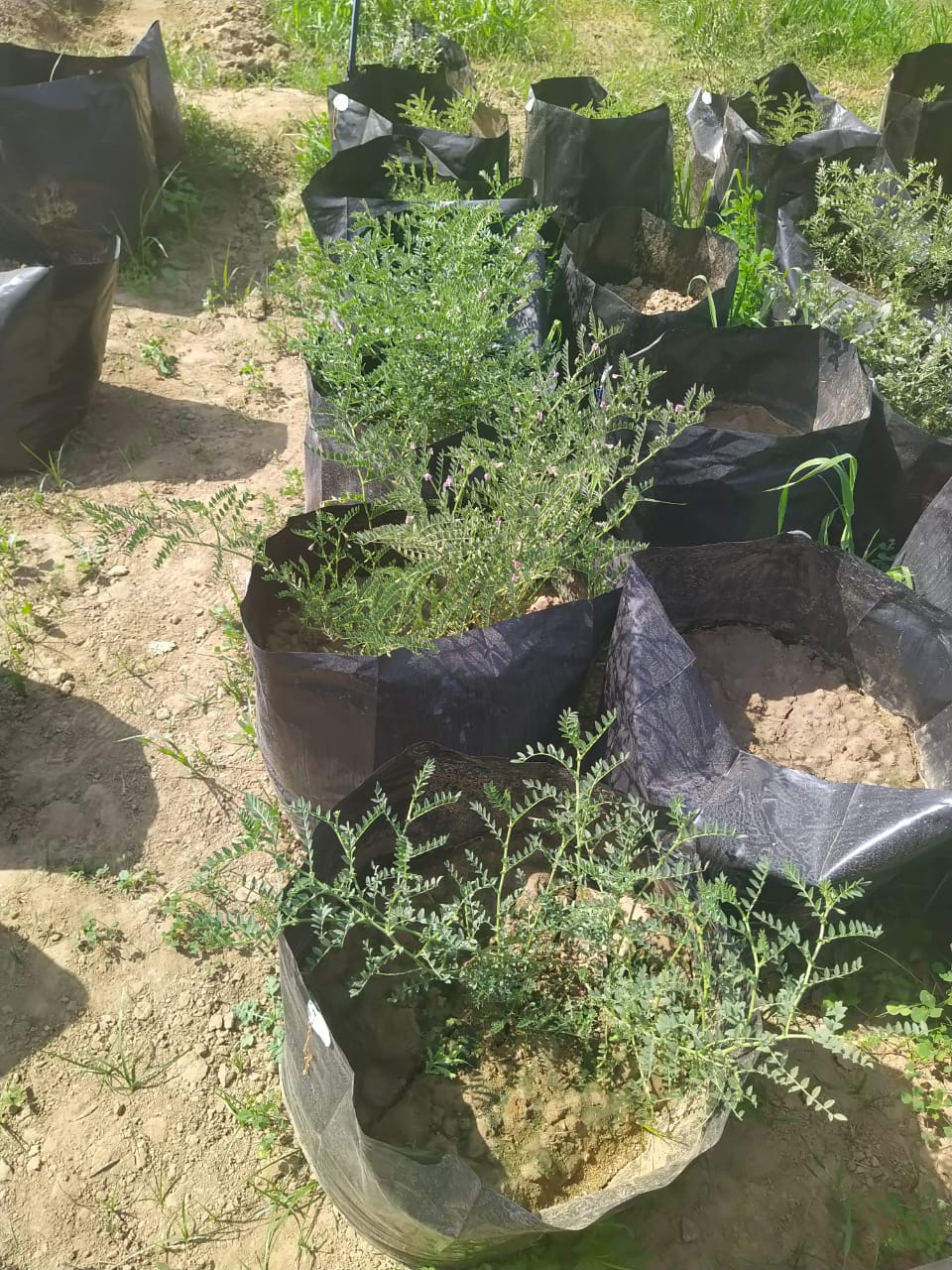

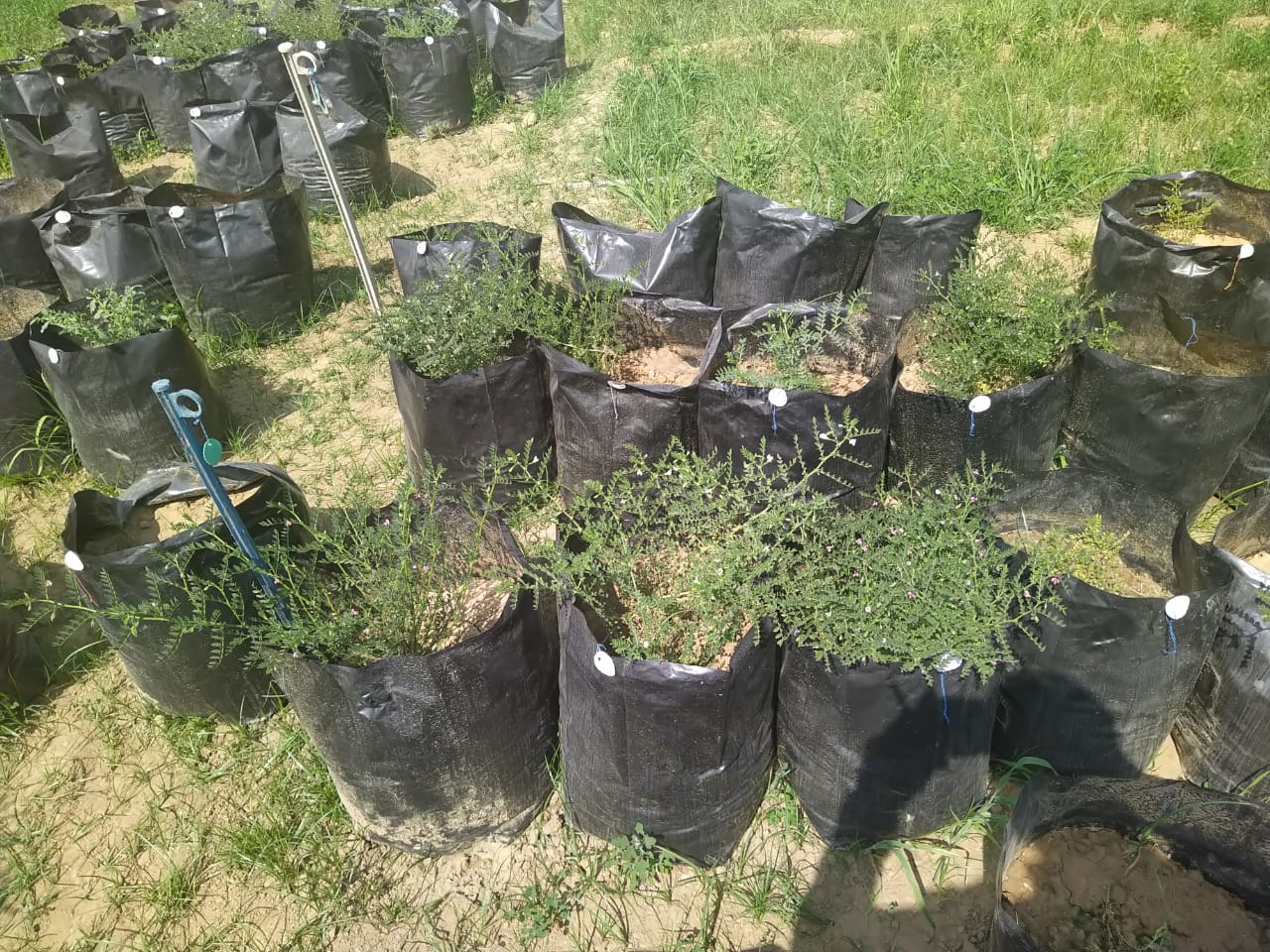


**b**

**Supplementary Figure S3.** (a) Initiation of experiment under rain-out shelter (ROS) during Rabi 2020 (Nov. 2020). The distance between the bags and the number of bags does not depict the actual distance and number, respectively. (b) Plant growth at 93 days after sowing (DAS) (Feb. 2021).


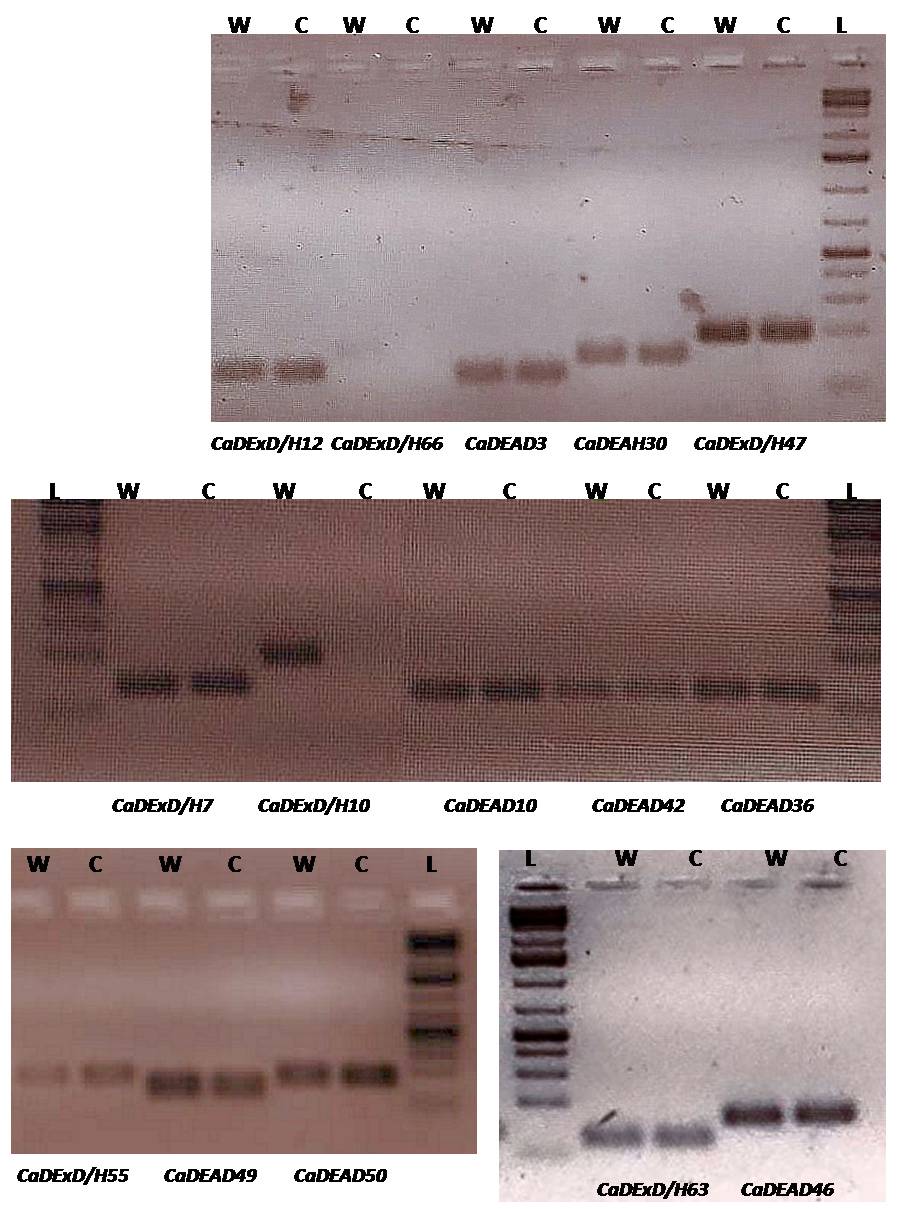


**Supplementary Figure S4.** The specificity of amplified products obtained after qRT-PCR amplification with the primers designed for amplification of the RNA helicase genes in the cDNA samples. The products were resolved on a 1.5% agarose gel (with EtBr); L: 100 bp plus DNA ladder; W : ILWC 292 (wild, *C. reticulatum*); C : ICC 8261 (kabuli, cultivated, *C. arietinum*).


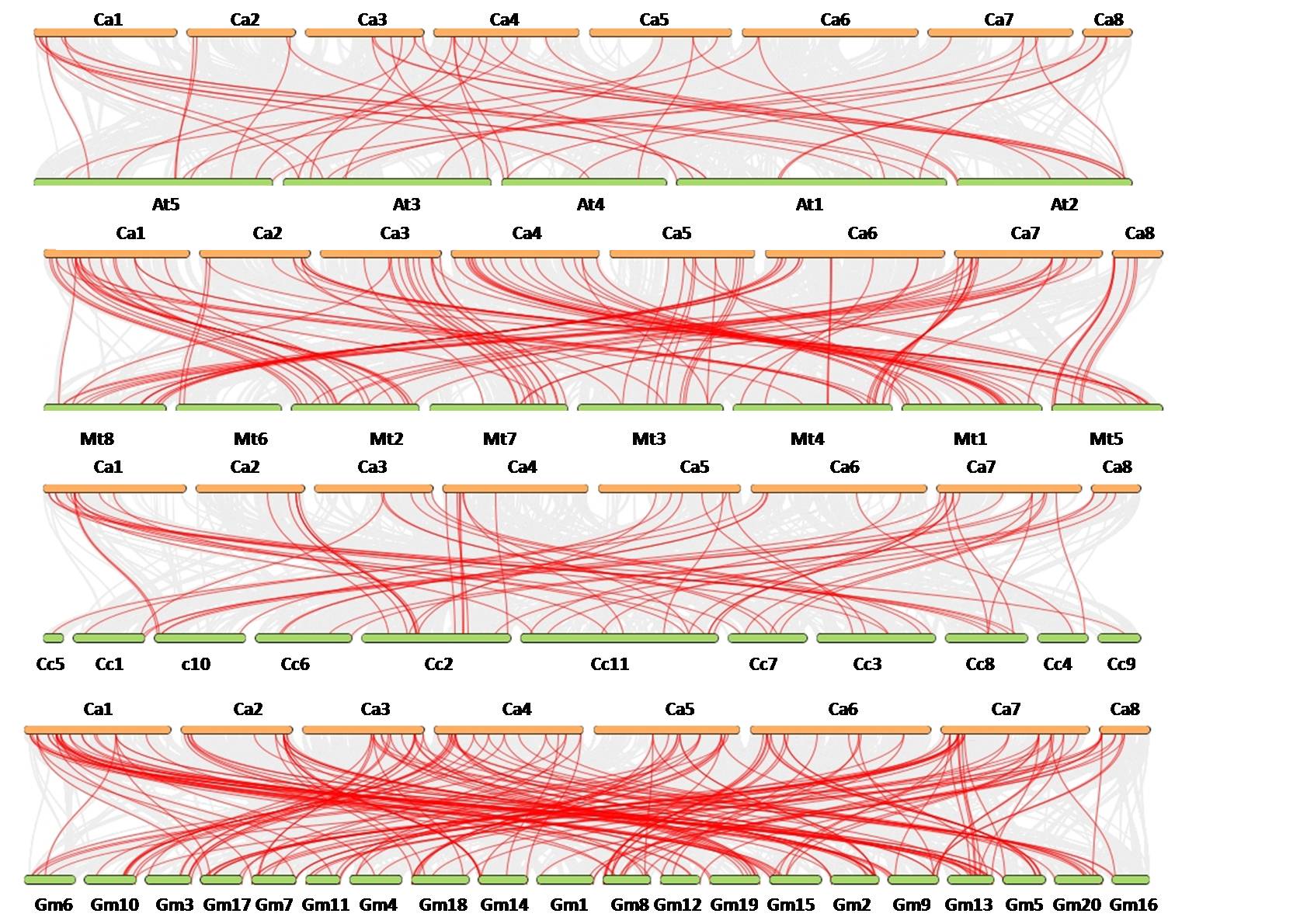


# Supplementary Figure S5. Synteny analysis of the RNA helicase genes between chickpea (Ca : *Cicer arietinum*) and other plant species (At: *Arabidopsis thaliana*; Mt : *Medicago truncatula*; Cc : *Cajanus cajan* and Gm : *Glycine max*). The gray lines in the background indicate the collinear blocks located on the chromosomes within the chickpea and other genomes, while the red lines indicate the syntenic RNA helicase gene pairs between the two genomes.


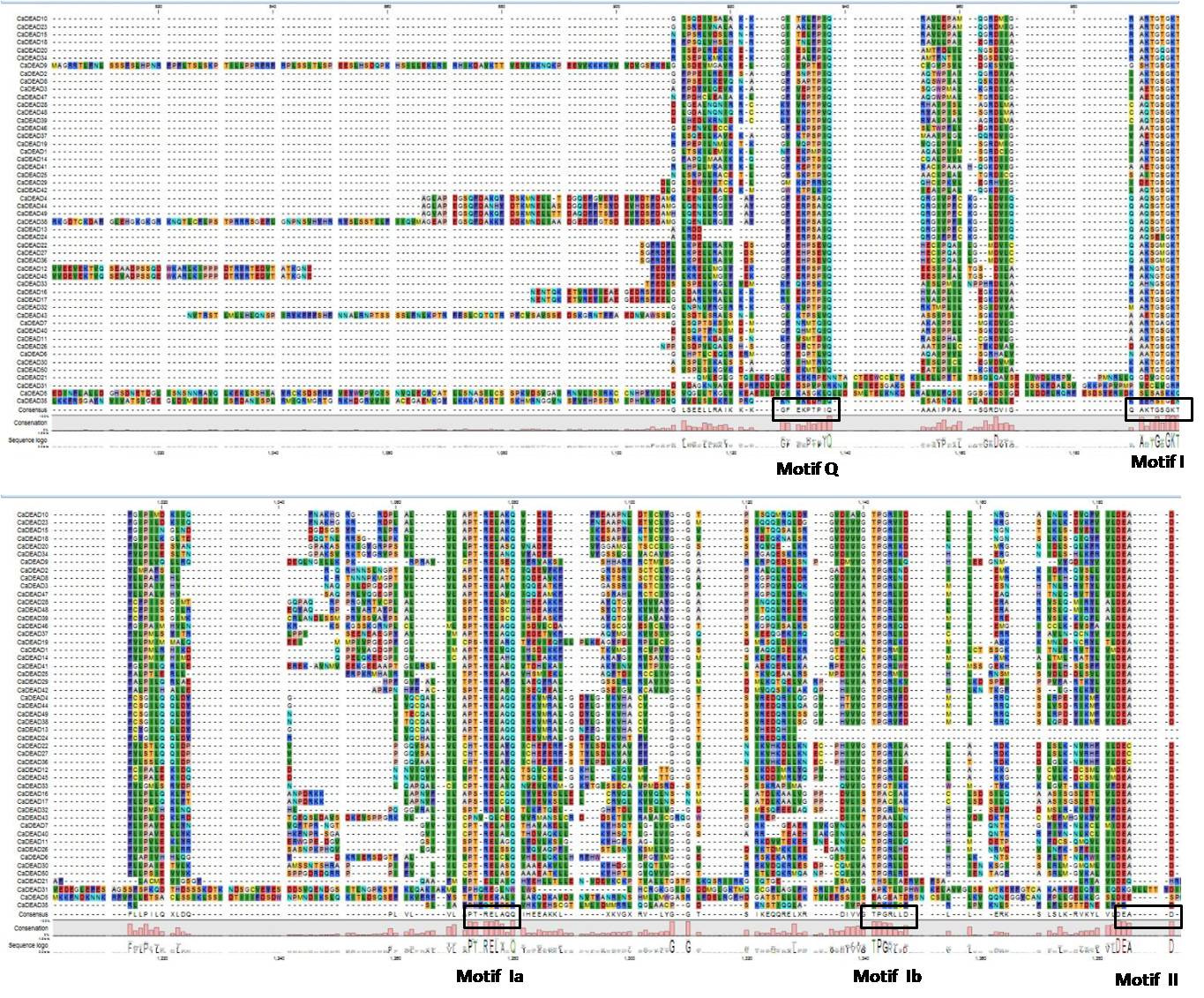


continued


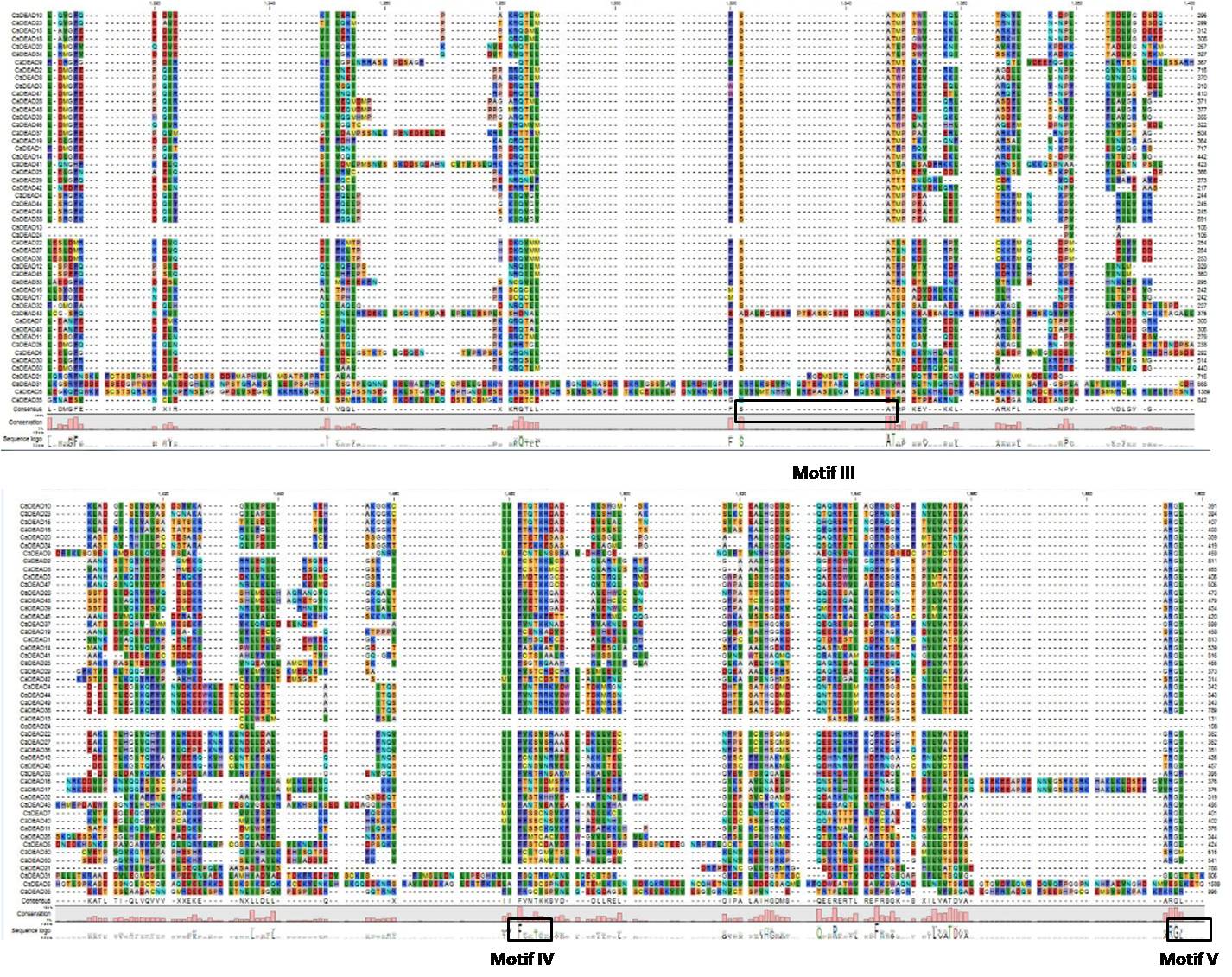


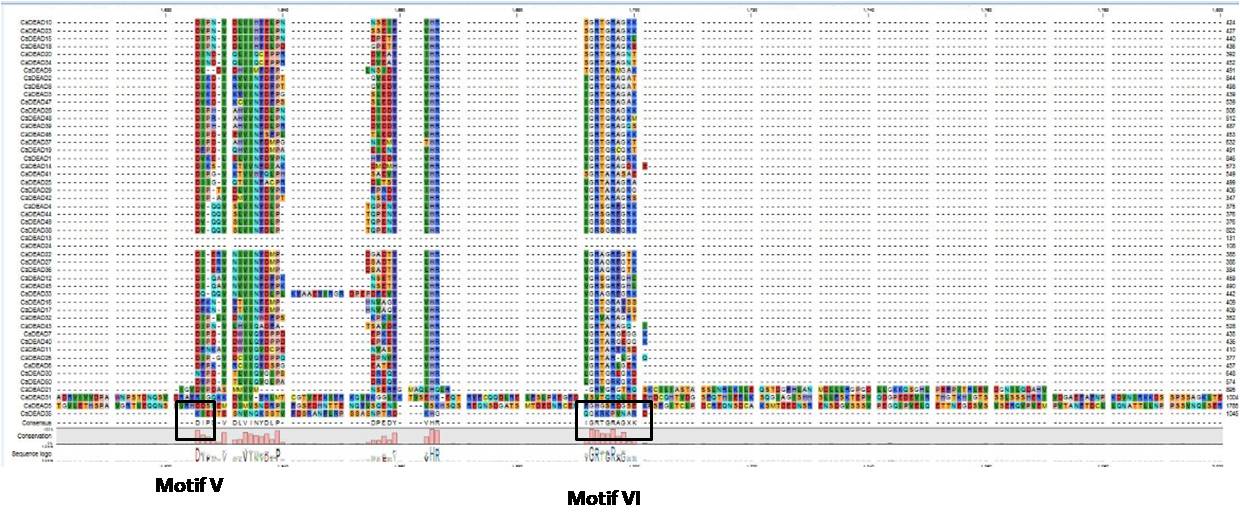


**Supplementary Figure S6.** Multiple sequence alignment (MSA) of the 50 CaDEAD-box proteins depicting the consensus sequences (enclosed in boxes) present at each of the 9 conserved motifs (Q, I, Ia, Ib, II, III, IV, V and VI) at the helicase core of the proteins. The lengths of the bars at the bottom represent the percent conservation at each amino acid position.


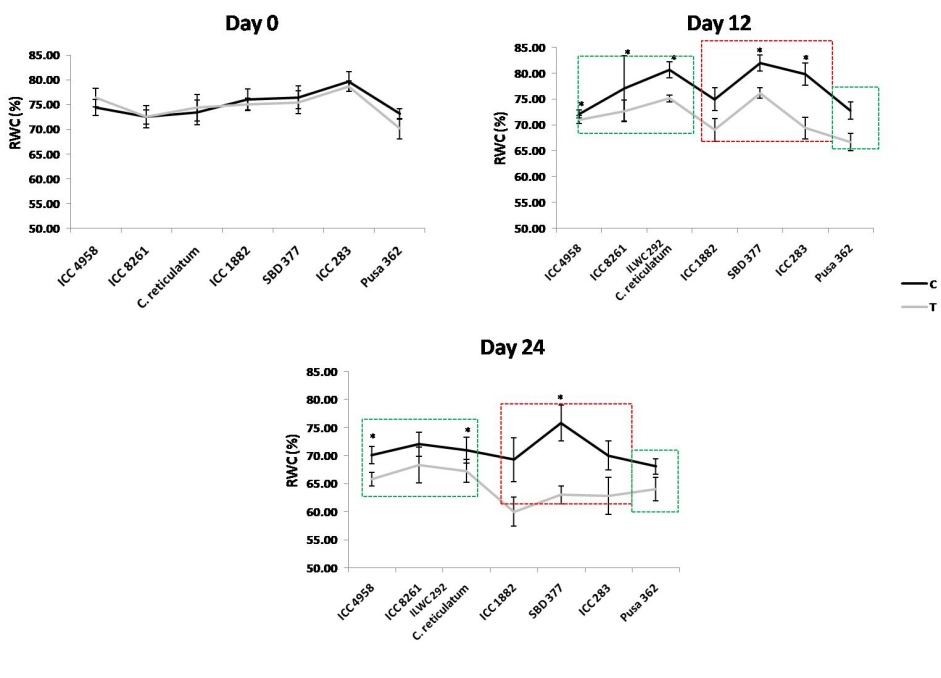


**a**


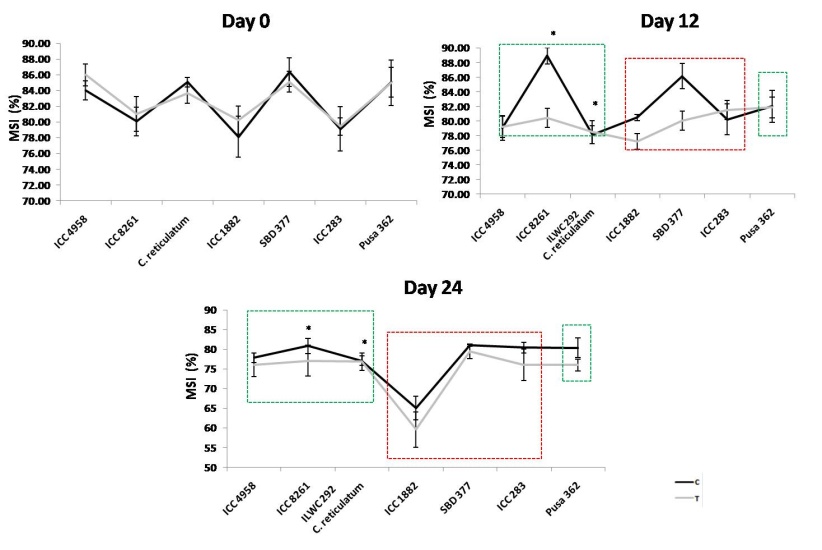


**b**

**Supplementary Figure S7.** (a) The changes in leaf relative water content (RWC) between the control (C) or well watered plants and drought stress treated plants (T), for the seven genotypes used in the study, namely ICC 4958, ICC 8261, ILWC 292, ICC 1882, SBD 377, ICC 283 and Pusa 362. ILWC 292 belongs to the wild species *C. reticulatum*, while the remaining six genotypes belong to the cultivated chickpea species, *C. arietinum*. The data for RWC was recorded on day 0 (93 DAS, initiation of drought stress treatment); day 12 (93+12=105 DAS) and day 24 (93+24=117 DAS). An average value of five biological replicates/genotype/treatment (n=5) were used for both the traits, to plot the line graphs. Vertical lines represent the mean ± SE. Statistical significance of differences in means, was tested by Student’s t-test. The * symbol represents significant difference at p < 0.05 between the means of C and T plants for a genotype. The green boxes depict reduction in mean values for the DT genotypes while red boxes depict reduction in mean values for the DS genotypes. (b) The changes in membrane stability index (MSI) between the control (C) or well watered plants and drought stress treated plants (T), for the seven genotypes. Sampling intervals remain the same as for RWC estimation.


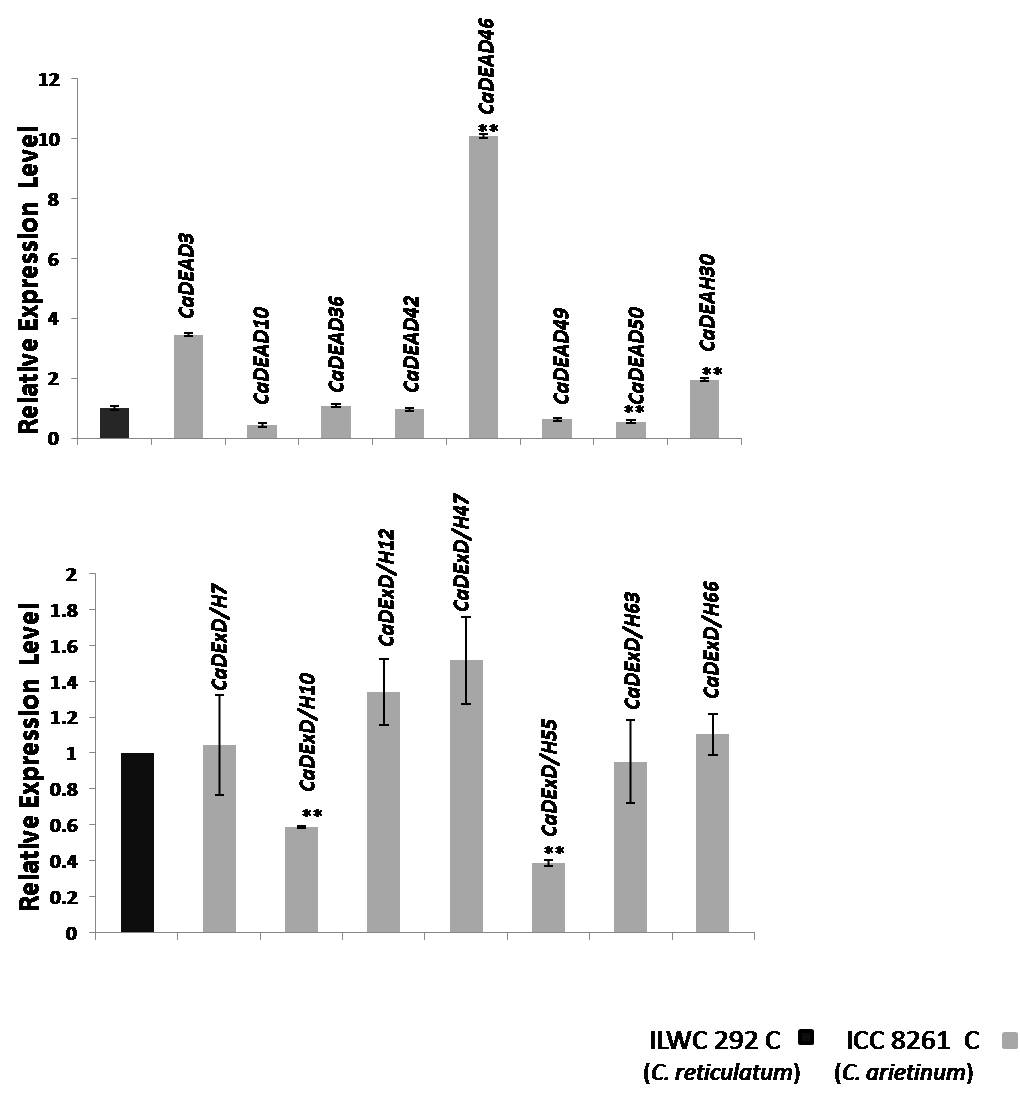


**a**

continued


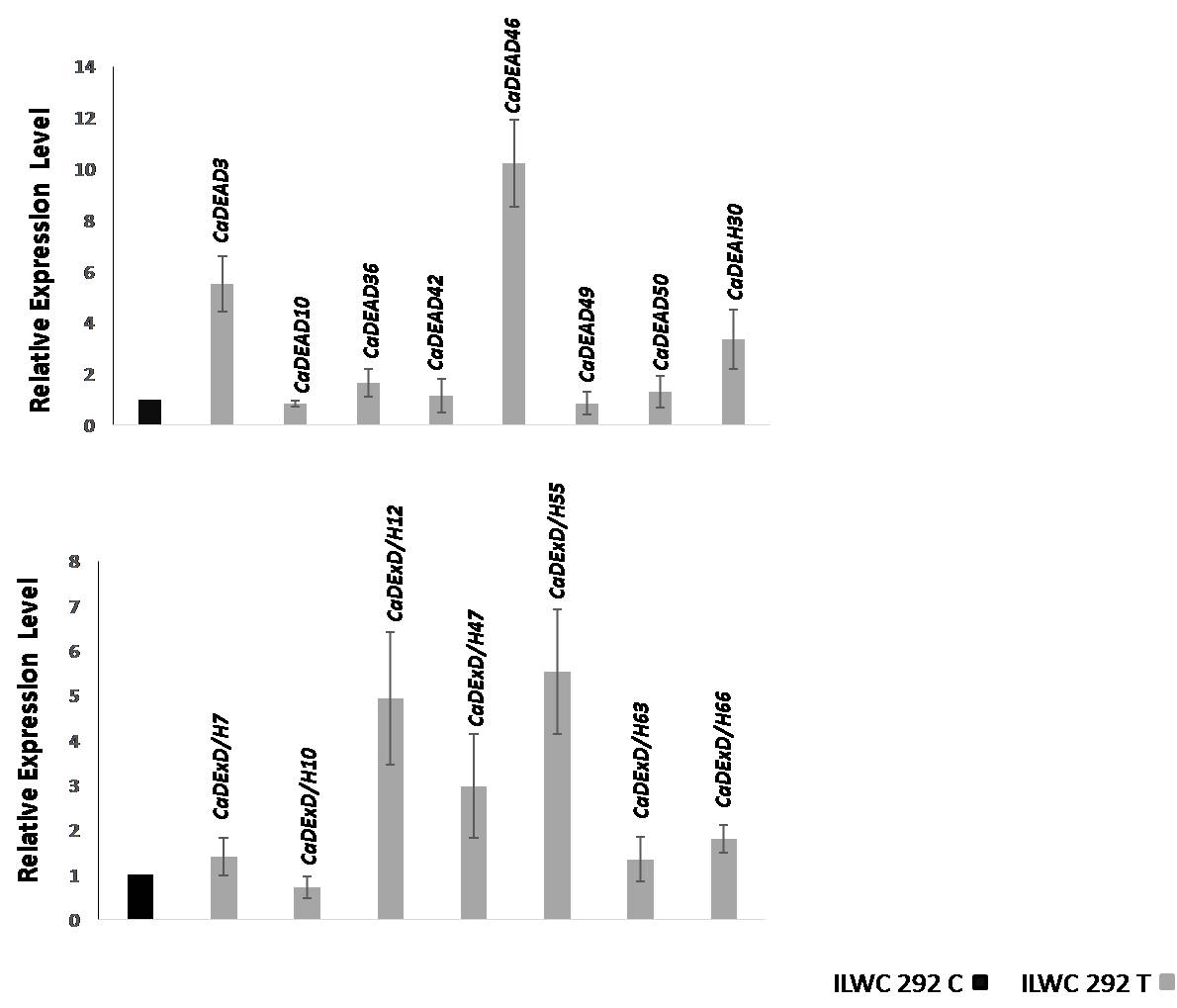


**b**

continued


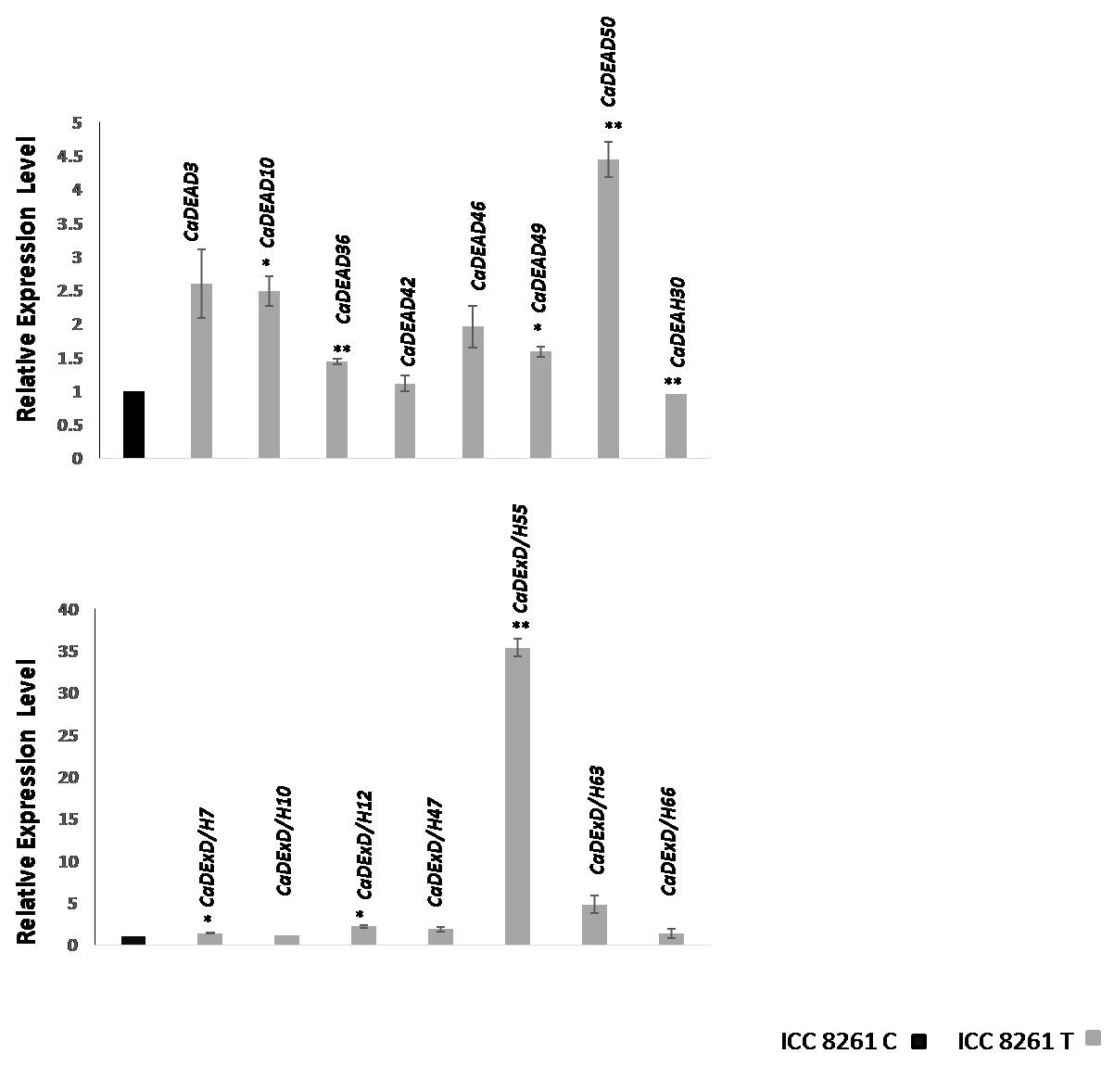


**c**

**Supplementary Figure S8.** The relative expression levels of selected fifteen RNA helicase genes (7 *CaDEAD*, 1 *CaDEAH* and 7 *CaDExD/H*- box genes) in leaf tissues of two genotypes, ILWC 292 (*C. reticulatum*) and ICC 8261 (*C. arietinum*), as estimated by qRT-PCR. All samples were collected on 117 DAS. The relative expression levels which were normalized to *GAPDH* were determined by the comparative CT method (2−ΔΔCT). Three biological and technical replicates were used for each experiment. Error bars indicate standard errors of means. The * symbol above bars indicates significant difference at p < 0.05 and ** indicates significant difference at p < 0.01. (a) The expression level of genes for ILWC 292 control (C24) or well watered plants was considered as control (expression = 1). The FC in gene expression levels of ICC 8261 C24 plants were estimated over the ILWC 292 C24 plants. (b) The expression level of genes for ILWC 292 C24 plants were considered as control (expression = 1) and the FC in gene expression levels of ILWC 292 T24 plants were estimated over the ILWC 292 C24 plants. (c) The expression level of genes for ICC 8261 C24 plants were considered as control (expression = 1) and the FC in gene expression levels of ICC 8261 T24 plants were estimated over the ICC 8261 C24 plants.

**Supplementary Table S1**. Details of primers used for qRT-PCR.

(Provided as a separate MS Excel file, sheet 1)

**Supplementary Table S2**. Statistical analysis of mean trait values for RWC, MSI and root traits for the seven genotypes, under control (C) and drought stress conditions (T). Means followed by different letters within a column are significantly different from each other at p < 0.05.

(Provided as a separate sheet in the above mentioned Excel file)

**Supplementary Table S3.** Details of the RNA helicase genes identified in chickpea genome.

(Provided as a separate sheet in the above mentioned Excel file)
